# Intrawound vancomycin for prevention of surgical site infection: meta-analysis of randomized clinical trials

**DOI:** 10.1093/bjsopen/zrag131

**Published:** 2026-09-21

**Authors:** Shaan Patel, Shiva A Nischal, Kush M Kale, Angelette Mendonca, Nelson Sofoluke, Srinivas K Prasad, James S Harrop, Daniel Refai

**Affiliations:** Department of Neurological Surgery, Thomas Jefferson University Hospital, Philadelphia, Pennsylvania, USA; Department of Physiology, Anatomy and Genetics, Medical Sciences Division, University of Oxford, Oxford, UK; Department of Physiology, Anatomy and Genetics, Medical Sciences Division, University of Oxford, Oxford, UK; School of Clinical Medicine, University of Cambridge, Cambridge, UK; Department of Neurological and Orthopedic Surgery, Emory University Hospital Midtown, Atlanta, Georgia, USA; Department of Neurological Surgery, Thomas Jefferson University Hospital, Philadelphia, Pennsylvania, USA; Department of Neurological Surgery, Thomas Jefferson University Hospital, Philadelphia, Pennsylvania, USA; Department of Neurological and Orthopedic Surgery, Emory University Hospital Midtown, Atlanta, Georgia, USA

**Keywords:** antimicrobial stewardship, antibiotic prophylaxis, gram-positive bacteria, local antibiotic delivery

## Abstract

**Background:**

Surgical site infection remains a major cause of postoperative morbidity. Intrawound vancomycin has been proposed as an adjunct to standard prophylaxis, but its effectiveness across surgical settings remains uncertain. This systematic review and meta-analysis of randomized clinical trials aimed to evaluate the association between intraoperative intrawound vancomycin and surgical site infection in adults undergoing surgery.

**Methods:**

MEDLINE, Embase, and CENTRAL were searched from database inception to March 2026 for randomized clinical trials comparing intrawound vancomycin with standard care in adults undergoing operative procedures. Two reviewers independently performed screening, data extraction, and risk-of-bias assessment using the Cochrane Risk of Bias 2 tool. Random-effects meta-analyses were undertaken, with certainty of evidence rated using the Grading of Recommendations Assessment, Development and Evaluation framework. Primary outcomes were overall surgical site infection, deep surgical site infection, superficial surgical site infection, and Gram-positive surgical site infection.

**Results:**

Twenty-two trials enrolling 8424 patients (vancomycin 4228, control 4196) were included. Intrawound vancomycin was associated with a lower risk of overall surgical site infection (risk ratio 0.79, 95% confidence interval 0.64 to 0.99) and Gram-positive surgical site infection (risk ratio 0.53, 0.35 to 0.79), corresponding to numbers needed to treat of 82 and 78, respectively. No significant differences were observed for deep or superficial surgical site infection, Gram-negative infection, reoperation, wound complications, mortality, or length of hospital stay. The certainty of evidence was moderate.

**Conclusion:**

Intrawound vancomycin was associated with a modest reduction in surgical site infection, driven by reductions in Gram-positive infections. These findings support a selective, context-specific use rather than routine application across all procedures.

## Teaser

In this meta-analysis of 22 randomized clinical trials enrolling 8424 adults, intrawound vancomycin was associated with a lower risk of overall surgical site infection (risk ratio 0.79, 95% confidence interval 0.64 to 0.99) and Gram-positive surgical site infection (risk ratio 0.53, 0.35 to 0.79). No significant effect was observed for deep, superficial, or Gram-negative infection, and the certainty of evidence was moderate. These findings support a selective, context-specific role rather than routine use across all procedures.

## Introduction

Surgical site infection (SSI) remains one of the most consequential complications of operative care. Despite advances in perioperative protocols, SSI continues to be associated with prolonged hospital stay, reoperation, and increased healthcare utilization across a wide range of surgical specialties. Although bundled prevention strategies have reduced infection rates, further incremental gains have proven difficult to achieve consistently, particularly in higher-risk operative settings^1,2^.

Intrawound vancomycin has been proposed as an adjunctive strategy to reduce SSI. By delivering high local antibiotic concentrations directly to the surgical bed, this approach aims to enhance antibacterial activity while minimizing systemic exposure. This biological rationale is strongest for Gram-positive organisms^3,4^, which account for a substantial proportion of SSIs in orthopaedic and neurosurgical procedures. However, the extent to which this targeted microbiological effect translates into meaningful reductions in clinically defined SSI remains uncertain.

The clinical role of intrawound vancomycin must also be considered within the broader context of contemporary infection prevention and antimicrobial stewardship^3,5^. SSI prevention relies on coordinated, evidence-based perioperative practices, and the addition of local antibiotics represents an incremental intervention rather than a primary strategy. Moreover, expanding perioperative antibiotic exposure raises concerns regarding antimicrobial resistance and unintended shifts in pathogen distribution. As such, any potential benefit must be interpreted alongside its magnitude, consistency, and microbiological specificity.

Randomized trials evaluating intrawound vancomycin have reported heterogeneous results, with variation in surgical populations, dosing strategies, and outcome definitions. Previous syntheses have often included non-randomized studies, limiting causal inference and complicating interpretation of treatment effects. More recently, additional randomized clinical trials (RCTs)^6–15^ have expanded the available evidence base across multiple surgical settings, necessitating an updated synthesis restricted to randomized data. A systematic review and meta-analysis was therefore undertaken to evaluate the association between intraoperative intrawound vancomycin and SSI. Overall, deep, superficial, and organism-specific SSIs were examined with prespecified analyses to explore whether treatment effects differed across operative contexts.

## Methods

### Protocol and registration

This systematic review and meta-analysis was conducted and reported in accordance with Cochrane Handbook for Systematic Reviews of Interventions^16^ and the PRISMA statement^17^. A study protocol was registered *a priori* in the International Prospective Register of Systematic Reviews (PROSPERO) (CRD420261353457).

### Search strategy and screening

A comprehensive search of MEDLINE, Embase, and Cochrane Central Register of Controlled Trials (CENTRAL) was conducted from database inception to 3 March 2026. The search combined controlled vocabulary and free-text terms related to intrawound vancomycin and SSI. Full search strategies are provided in *Table S1*. Reference lists of included articles and previous reviews were also screened. Two reviewers independently carried out screening for title and abstract relevance, followed by full-text eligibility assessment. Disagreements were resolved through consensus. Inter-rater reliability between reviewers was excellent (Cohen’s κ = 0.94).

### Inclusion criteria

RCTs enrolling adult patients (aged ≥ 16 years) undergoing operative procedures in which intrawound vancomycin was administered during operation and compared with standard care were included. Studies were required to report at least one prespecified efficacy or safety outcome. Studies were excluded if they were non-randomized, included a single arm, or lacked outcome data (including trial registrations without results). Review articles, conference abstracts without full data sets, and non-human studies were also excluded.

### Data extraction

Data were collected independently by two reviewers using a predefined extraction template. Information obtained from each study included trial design characteristics (authors, year, country, enrolment period), sample size, details of the vancomycin intervention (dose, timing, formulation), operative context, duration of follow-up, and reported clinical outcomes. Any discrepancies between reviewers were resolved through consensus.

### Outcomes and subgroup analyses

The primary outcomes were overall SSI, deep SSI, superficial SSI, and SSI attributable to Gram-positive organisms.

Secondary outcomes included infections due to Gram-positive cocci, methicillin-resistant *Staphylococcus aureus* (MRSA), and Gram-negative organisms, as well as reoperation, abscess, wound dehiscence, mortality, and length of hospital stay (LOS). Subgroup analyses were prespecified to explore variation in treatment effect according to vancomycin dose (1 g, 1–2 g, 2 g), use of instrumentation (instrumented, non-instrumented, mixed), surgical site (lower limb, cranial, spinal, mandibular), surgical specialty (orthopaedic, neurosurgery, maxillofacial), and SSI definition.

### Risk of bias and certainty of evidence

Two reviewers independently assessed risk of bias, using the Cochrane Risk of Bias 2 tool^18^, and certainty of evidence, using the Grading of Recommendations Assessment, Development and Evaluation framework^19^, for all included trials. Disagreements were resolved by consensus with senior adjudication when required. Publication bias was assessed by means of visual inspection of funnel plots and Egger’s regression testing when sufficient studies (≥ 10) were available.

### Statistical analysis

All statistical analyses were carried out using R version 4.4.2 (R Project for Statistical Computing, Vienna, Austria)^20^. Meta-analyses were performed using random-effects models with restricted maximum likelihood estimation to account for interstudy heterogeneity. Effect estimates were expressed as risk ratios (RRs) for dichotomous outcomes and weighted mean differences (MDs) for continuous outcomes, with 95% confidence intervals; odds ratios were used in rare-event sensitivity analyses. Interstudy statistical heterogeneity was assessed using Cochran’s Q test and quantified using *I*^2^ statistics (> 25% suggesting meaningful heterogeneity). The robustness of findings was assessed using leave-one-out, sparse-event and low-risk-of-bias sensitivity analyses. Study influence and contribution to heterogeneity were further evaluated using Baujat plots. Trial sequential analysis (TSA) was undertaken for selected outcomes to assess the sufficiency of cumulative evidence and adjust for potential random error (2-sided α = 0.05; power 90%). Exploratory meta-regression analyses were conducted to evaluate whether study-level characteristics (age, sex distribution, body mass index (BMI), diabetes prevalence, smoking status, operating time, use of instrumentation, vancomycin dose, control-group SSI risk) were associated with variation in treatment effects. Absolute risk differences were calculated for selected outcomes, from which the number needed to treat (NNT) for benefit and the number needed to harm were calculated.

## Results

### Study identification and cohort characteristics

**Figure 1:**
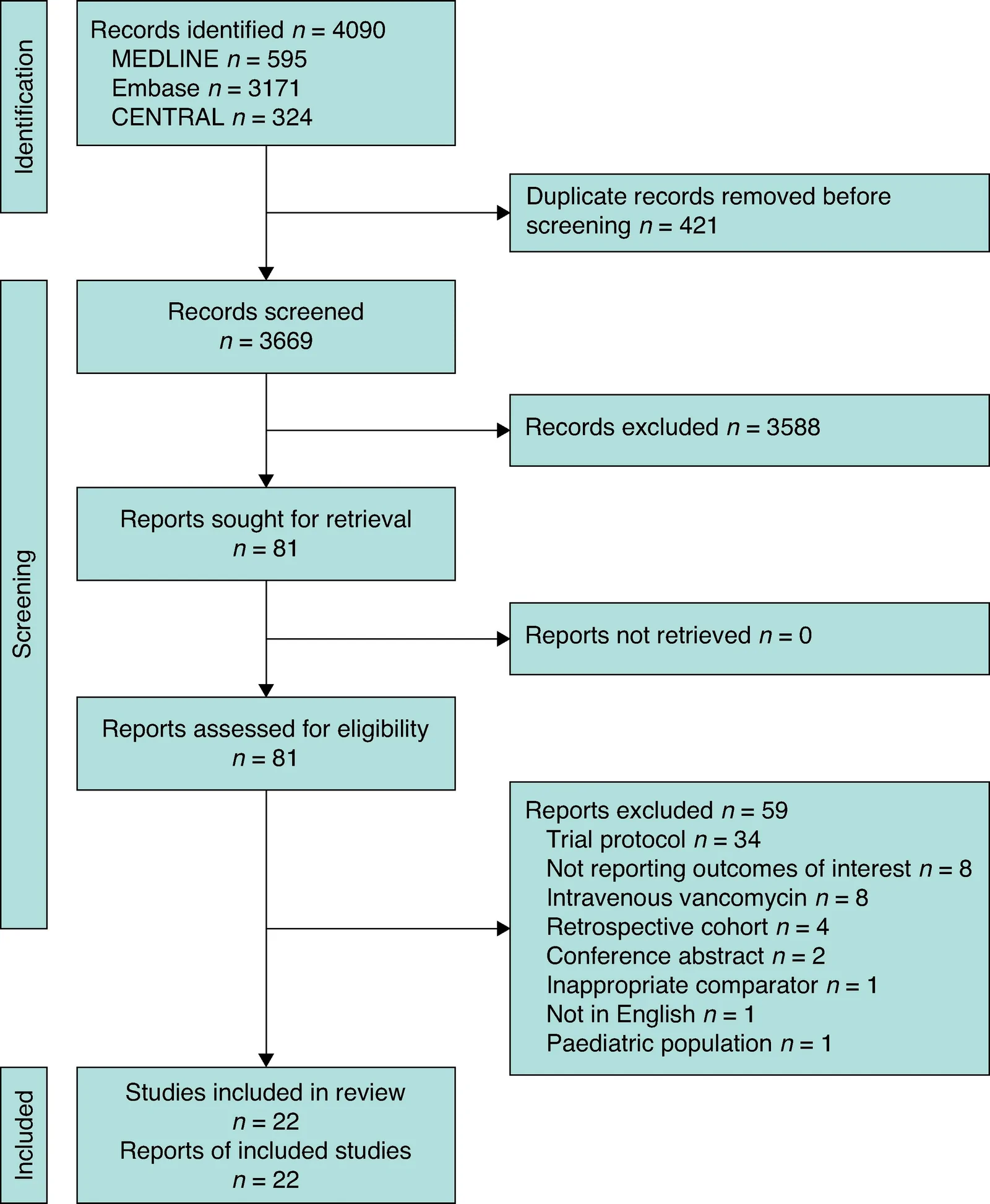
PRISMA flow diagram showing selection of articles evaluating intrawound vancomycin for review

The search yielded 4090 records, of which 22 RCTs^4,6–15,21–31^ comprising 8424 patients (vancomycin 4228, control 4196) met the inclusion criteria (*Fig. 1*). Included studies spanned multiple operative settings, including orthopaedic (8 studies), spinal (9 studies), cranial (2 studies), maxillofacial (1 study), and mixed craniospinal (2 studies) procedures. Trial sample sizes ranged from 21 to 552 patients, with mean patient age (range 31.9–74.0 years) and proportion of male patients (29.0–91.0%) varying widely across studies (*Table 1*). Intervention details are summarized in *Table 2*. Detailed study characteristics are provided in *Tables S2–S8*.

**Table 1:** Baseline characteristics of included randomized clinical trials evaluating intrawound vancomycin across orthopaedic, spinal, cranial, and maxillofacial procedures

Reference	Country	Enrolment period (years)	Surgical procedure	No. of patients		Age (years)*		Male patients (%)	
				Vancomycin	Control	Vancomycin	Control	Vancomycin	Control
Abuzaiter *et al.*^21^ (2023)	Canada	2016–2017	Total knee arthroplasty	80	85	66.0	64.0	41.3	34.1
Acosta-Olivo *et al.*^14^ (2024)	Mexico	n.r.	ORIF for ankle fractures	66	66	37.7(14.6)	40.1(13.0)	49.2	48.5
Atallah *et al.*^22^ (2021)	Egypt	2020	Cranial surgery	39	47	32.1(15.9)	31.9(16.7)	87.2	83
do Nascimento *et al.*^23^ (2020)	Italy	2014–2018	Instrumented spinal surgery	49	47	43.0(14.9)	45.0(15.0)	81.6	66.0
Gandhi *et al.*^24^ (2024)	India	2021–2022	ORIF for closed fractures	44	44	40.4(14.2)	40.9(15.6)	81.8	65.9
Han *et al.*^11^ (2025)	China	2021–2022	Instrumented spinal surgery	78	78	59.3(13.3)	62.9(8.6)	44.0	45.0
Hasan *et al.*^25^ (2020)	Iraq	2015–2019	Non-instrumented spinal surgery	192	198	38.2(8.1)	34.7(7.2)	91.2	69.2
Kannan *et al.*^15^ (2026)	USA	2014–2019	Cranial and non-instrumented spinal surgery	552	551	54.2(16.0)	53.9(16.5)	42.9	44.1
Khalid *et al.*^26^ (2023)	Pakistan	2019–2021	Instrumented spinal surgery	39	39	36.0(16.6)	33.7(15.9)	53.8	58.9
Kunakornsawat *et al.*^27^ (2019)	Thailand	2013–2015	Instrumented spinal surgery	265	135	52.1(14.5)	55.2(14.5)	42.6	36.3
Mirzashahi *et al.*^28^ (2018)	Iran	2014–2015	Instrumented and non-instrumented spinal surgery	193	187	42.1(20.1)	61.4(9.4)	35.5	
Mohammadhoseini *et al.*^6^ (2024)	Iran	2023	Hip hemiarthroplasty	24	24	72.0(5.3)	74.0(3.5)	41.6	41.6
Morgan *et al.*^12^ (2024)	Nigeria	2021–2022	Cranial and non-instrumented spinal surgery	21	21	48.1(17.1)	46.0(19.1)	66.7	85.7
Mulpur *et al.*^13^ (2024)	India	2021–2022	Total knee arthroplasty	507	515	61.7(7.5)	61.4(7.4)	29.0	30.5
O’Toole *et al.*^4^ (2021)	USA	2015–2017	ORIF for tibial fractures	481	499	45.4(14.0)	46.1(13.5)	62.2	63.7
Rathore *et al.*^10^ (2025)	India	2024–2025	ORIF for long-bone fractures	64	64	44.1(3.4)	43.2(12.5)	78.1	71.9
Saba *et al.*^9^ (2025)	USA	n.r.	Total hip arthroplasty and total knee arthroplasty	455	484	66.1(11.6)	66.2(11.5)	35.4	36.2
Salimi *et al.*^29^ (2022)	Iran	2018–2019	Instrumented and non-instrumented spinal surgery	187	188	51.7	52.3	42.2	44.7
Singh *et al.*^30^ (2019)	India	2017	ORIF for mandibular fractures	50	50	32.5	33.2	80.0	84.0
Tubaki *et al.*^31^ (2013)	India	2011–2012	Instrumented and non-instrumented spinal surgery	433	474	44.2(18.9)	46.6(19.6)	54.1	57.7
Wang *et al.*^7^ (2025)	Taiwan	2017–2023	Instrumented spinal surgery	357	348	68.1(11.3)	67.3(10.7)	36.7	37.4
Yusuf *et al.*^8^ (2026)	Nigeria	2022–2023	Cranial surgery	52	52	40.0(12.8)	42.0 14.2)	59.6	63.5

^*^Values are mean(standard deviation). n.r., Not reported; ORIF, open reduction and internal fixation.

**Table 2:** Intervention details of included randomized controlled trials

Reference	Vancomycin dose (g)	Standard antibiotic prophylaxis	Surgical site infection definition
Abuzaiter *et al.*^21^ (2023)	1	Cefazolin 2–3 g i.v. before surgery	MSIS criteria
Acosta-Olivo *et al.*^14^ (2024)	1	Cefalotin 1 g i.v. before surgery, followed by cefalotin 1 g i.v. every 8 h for 24 h after surgery	CDC criteria
Atallah *et al.*^22^ (2021)	1	Cefazolin i.v. within 30 min before incision; continued for 48 h after surgery	Culture-positive wound infection within 120 days of surgery
do Nascimento *et al.*^23^ (2020)	2	Standard antibiotic prophylaxis (regimen non-specified)	CDC criteria
Gandhi *et al.*^24^ (2024)	2	Ceftriaxone 1 g i.v. at incision, followed by ceftriaxone 1 g i.v. every 8 h for 24 h after surgery	International consensus definition of fracture-related infection
Han *et al.*^11^ (2025)	1	Cefuroxime 1.5 g i.v. within 1 h before incision; repeated if duration of operation exceeded 3 h	CDC criteria
Hasan *et al.*^25^ (2020)	1–2	Cefazolin 1 g i.v. 1 h before surgery, followed by 2 postoperative doses of cefazolin 1 g i.v. 12 h apart	Clinical evidence of infection
Kannan *et al.*^15^ (2026)	2	Cefazolin i.v. 1 h before incision	Readmission or positive wound culture
Khalid *et al.*^26^ (2023)	1	Cefoperazone–sulbactam 2 g i.v. within 30 min before incision, followed by cefoperazone–sulbactam 1 g i.v. every 12 h for 3 days after surgery	Infection occurring within 3 months after surgery requiring additional operation and antibiotic treatment
Kunakornsawat *et al.*^27^ (2019)	1–2	Cefazolin 1 g i.v. within 60 min before incision, followed by oral dicloxacillin or clindamycin for 7 days after drain removal	CDC criteria
Mirzashahi *et al.*^28^ (2018)	1–2	Cefazolin 1 or 2 g i.v. 20 min before incision; repeated every 3 h during surgery or earlier if blood loss > 1000 ml	CDC criteria
Mohammadhoseini *et al.*^6^ (2024)	1	Cefazolin 2 g i.v. 30 min before skin incision	MSIS criteria
Morgan *et al.*^12^ (2024)	1	Ceftriaxone 1 g i.v. at induction of anaesthesia	CDC criteria
Mulpur *et al.*^13^ (2024)	2	Cefuroxime 1.5 g i.v. for 3 perioperative doses	MSIS criteria
O’Toole *et al.*^4^ (2021)	1	Systemic antibiotic prophylaxis at definitive fixation	CDC criteria
Rathore *et al.*^10^ (2025)	1	Standard antibiotic prophylaxis (regimen non-specified)	Infection within 30 days of surgery (or 1 year if implant)
Saba *et al.*^9^ (2025)	2	Standard antibiotic prophylaxis (regimen non-specified)	International Consensus Meeting criteria for periprosthetic joint infection
Salimi *et al.*^29^ (2022)	1–2	Cephalosporin 2 g i.v. within 2 h before surgery; repeated if duration of operation > 2 h. After operation, cephalosporin 1 g i.v. every 8 h for 24 h	CDC criteria
Singh *et al.*^30^ (2019)	1	Standard antibiotic prophylaxis (regimen non-specified)	Clinical evidence of infection
Tubaki *et al.*^31^ (2013)	1	Cefuroxime 750 mg i.v. before incision, followed by cefuroxime 750 mg i.v. every 8 h for 1 day or until drain removal	Clinical evidence of infection
Wang *et al.*^7^ (2025)	1–2	Cephalosporin 1 g i.v. 30 min before surgery; repeated every 4 h during surgery or if blood loss > 1000 ml. After operation, cephalosporin 1 g i.v. every 8 h plus aminoglycoside 80 mg every 12 h for 3 days	CDC criteria
Yusuf *et al.*^8^ (2026)	1	Ceftriaxone i.v. before surgery	CDC criteria

i.v., Intravenous; MSIS, Musculoskeletal Infection Society; h, hours; min, minutes; CDC, Centers for Disease Control and Prevention.

### Primary outcomes

**Figure 2:**
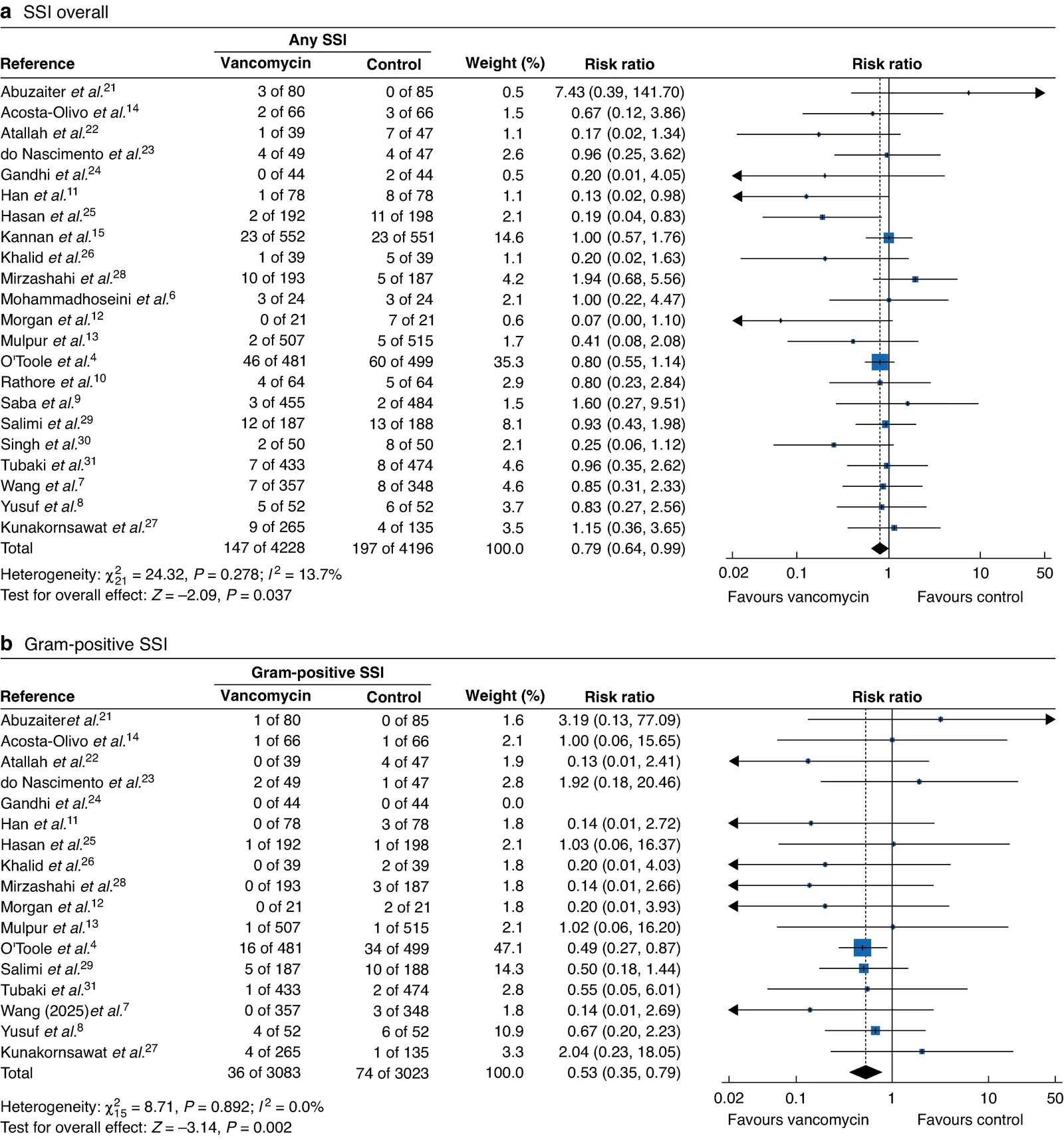
Forest plots showing impact of intrawound vancomycin on overall and Gram-positive SSI **a** Overall proportion of patients experiencing surgical site infection (SSI); **b** proportion of patients experiencing SSI due to Gram-positive organisms. Random-effects meta-analyses were undertaken using the restricted estimated maximum likelihood method. Risk ratios are shown with 95% confidence intervals. Squares represent study-specific effect estimates with sizes proportional to study weight; diamonds represent pooled estimates.

**Figure 3:**
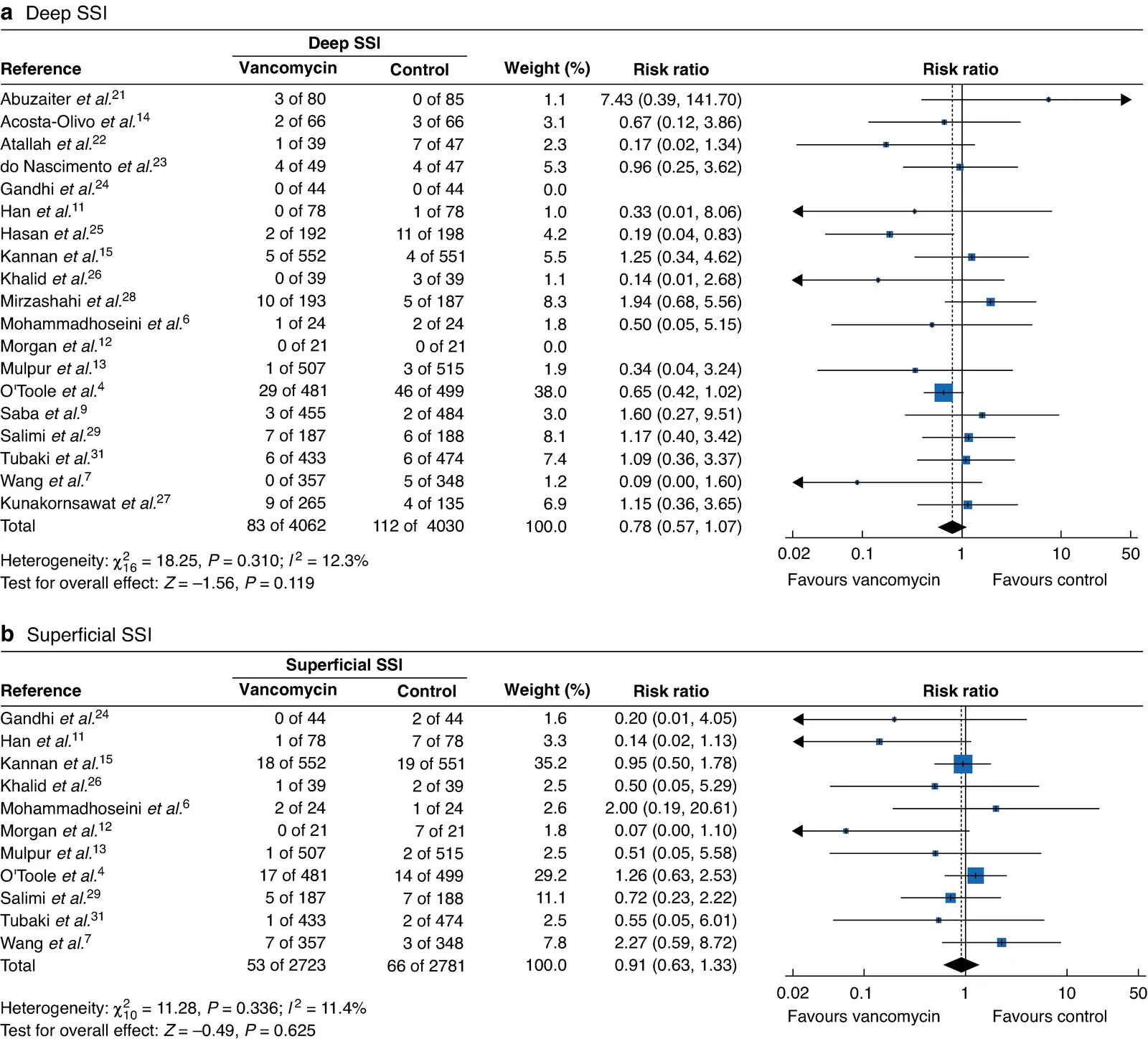
Forest plots showing impact of intrawound vancomycin on deep and superficial SSI **a** Proportion of patients experiencing deep surgical site infection (SSI); **b** proportion of patients experiencing superficial SSI. Random-effects meta-analyses were undertaken using the restricted estimated maximum likelihood method. Risk ratios are shown with 95% confidence intervals. Squares represent study-specific effect estimates with sizes proportional to study weight; diamonds represent pooled estimates.

Intrawound vancomycin was associated with a lower risk of overall SSI (RR 0.79, 95% confidence interval (c.i.) 0.64 to 0.99; *P* = 0.037; *I*^2^ = 13.7%)^4,6–15,21–31^ and Gram-positive SSI (RR 0.53, 0.35 to 0.79; *P* = 0.002; *I*^2^ = 0.0%)^4,7,8,11–14,21–29,31^ (*Fig. 2*). There was no significant difference for deep SSI (RR 0.78, 0.57 to 1.07; *P* = 0.119; *I*^2^ = 12.3%)^4,6,7,9,11–15,21–29,31^ or superficial SSI (RR 0.91, 0.63 to 1.33; *P* = 0.625; *I*^2^ = 11.4%)^4,6,7,11–13,15,24,26,29,31^ (*Fig. 3*).

### Absolute effects

Absolute risk differences corresponded to a NNT of 82 for overall SSI and 78 for Gram-positive SSI, indicating that approximately 1 infection may be prevented for every 78–82 patients treated.

### Secondary outcomes

Intrawound vancomycin was associated with a significant reduction in SSI due to Gram-positive cocci (RR 0.57, 95% c.i. 0.33 to 0.98; *P* = 0.043; *I*^2^ = 0.0%)^7,8,11–14,21–29,31^, but not MRSA (RR 0.18, 0.03 to 1.00; *P* = 0.050; *I*^2^ = 0.0%)^4,7,8,11–14,21–29,31^ or Gram-negative organisms (RR 1.02, 0.62 to 1.66; *P* = 0.953; *I*^2^ = 0.0%)^4,7,8,11–14,21–29,31^ (*Figs S1–S3*). No significant differences were observed for risk of reoperation (RR 0.84, 0.60 to 1.19; *P* = 0.328; *I*^2^ = 0.0%)^4,8,9,11,13,21,23,25,26,28,29,31^, abscess (RR 1.38, 0.73 to 2.62; *P* = 0.327; *I*^2^ = 0.0%)^9,13,15,25^, wound dehiscence (RR 0.83, 0.51 to 1.35; *P* = 0.449; *I*^2^ = 0%)^4,9,12^, mortality (RR 0.73, 0.30 to 1.80; *P* = 0.497; *I*^2^ = 20.1%)^7,11–13,15,22,23,25^, or LOS (MD −0.06 days, −1.05 to 0.93; *P* = 0.911; *I*^2^ = 75.0%)^7,23,30^ (*Figs S4–S8*).

### Sensitivity and influence analyses

Leave-one-out analyses demonstrated that the pooled estimate for Gram-positive SSI remained stable across study exclusions. In contrast, estimates for overall SSI and Gram-positive cocci showed some dependence on individual studies (*Figs S9–S11*). Sparse-event sensitivity analyses were directionally consistent across all primary outcomes and did not materially alter inference (*Table S9*). Findings were also directionally consistent in analyses restricted to trials at low risk of bias (*Table S10*) and restriction to strict vancomycin powder-only studies (*Table S11*). Baujat plots indicated that no single study disproportionately influenced pooled estimates for overall SSI, Gram-positive SSI, or superficial SSI. For deep SSI, however, the study by Mirzashahi *et al*.^28^ showed notably greater influence than other studies (*Figs S12–S15*).

### Subgroup analyses

No significant effect modification was observed across prespecified subgroup analyses by vancomycin dose, instrumentation status, surgical site, surgical specialty, or SSI definition (*Figs S16–S41*). Procedure-specific estimates for overall SSI stratified by surgical specialty, surgical site, and instrumentation status are shown in *Figs S25*, *S30*, and *S35*. By surgical specialty, the pooled RR was 0.82 (95% confidence interval 0.64 to 1.04) for orthopaedic, 0.57 (0.23 to 1.43) for neurosurgical, and 0.25 (0.06 to 1.12) for maxillofacial procedures. By instrumentation status, the RR was 0.78 (0.60 to 1.01) for instrumented and 0.59 (0.32 to 1.07) for non-instrumented procedures. No individual procedural subgroup reached statistical significance, and tests for subgroup differences were not significant. These stratified analyses were constrained by few trials per subgroup and were underpowered to confirm or exclude clinically meaningful procedure-specific differences.

### TSA findings

TSA demonstrated that the cumulative evidence for overall SSI and Gram-positive SSI crossed both monitoring boundaries and required information sizes, indicating sufficient evidence for these outcomes. In contrast, analyses for deep and superficial SSI did not reach required information sizes, suggesting insufficient power to exclude smaller treatment effects (*Figs S42–S45*).

### Meta-regression

Exploratory meta-regression showed that increasing mean age was associated with attenuation of the relative treatment effect of intrawound vancomycin on overall SSI (β = 0.028 (standard error (s.e.) 0.013) per year; *P* = 0.032), whereas a higher male proportion was associated with greater relative treatment benefit (β = −2.037 (s.e. 0.810); *P* = 0.012) (*Fig. S46* and *Table S12*). Higher control-group SSI risk was also associated with larger treatment effects (β = −0.056 (s.e. 0.023); *P* = 0.017) (*Fig. S46* and *Table S12*). No significant associations were observed for vancomycin dose, instrumentation status, diabetes prevalence, smoking, BMI, or operating time.

### Risk of bias and certainty of evidence

Of the 22 included trials, 14 were judged to have a low risk of bias and 8 had some concerns (*Table S13*). The certainty of evidence for all primary outcomes was rated as moderate (*Table S14*).

### Publication bias

Funnel plots showed no clear asymmetry (*Figs S47–S50*), and Egger’s regression tests demonstrated no evidence of small-study effects for overall SSI (*P* = 0.088), Gram-positive SSI (*P* = 0.994), deep SSI (*P* = 0.598), or superficial SSI (*P* = 0.067).

## Discussion

In this meta-analysis of 22 randomized trials including 8424 patients, intrawound vancomycin was associated with a modest reduction in overall SSI, driven primarily by reductions in Gram-positive infections. No clear differences were observed for deep or superficial SSI, and no signal was identified for Gram-negative infection. Heterogeneity across primary analyses was low, and treatment effects were consistent across prespecified groups. These findings suggest that the benefit of intrawound vancomycin is selective rather than global, reflecting antimicrobial specificity rather than a generalized reduction in postoperative infection risk.

The magnitude of effect was modest. The NNT was 82 to prevent 1 overall SSI and 78 for 1 Gram-positive SSI. These estimates indicate that intrawound vancomycin functions as an adjunctive rather than transformative intervention. Its clinical value is therefore likely to be context-dependent, with greater relevance in procedures with raised baseline infection risk or where the consequences of SSI, particularly implant-associated infection, are substantial^32,33^. This interpretation aligns with contemporary fracture literature, including the VANCO trial^4^ of surgically treated tibial plateau and pilon fractures, in which intrawound vancomycin reduced deep infection by approximately 3–4%, driven by reductions in Gram-positive infection without an effect on Gram-negative pathogens.

The divergence between organism-specific and anatomical outcomes provides important mechanistic insight. The most consistent signal was observed for Gram-positive SSI, whereas pooled estimates for deep and superficial infection were attenuated and crossed the null. This pattern is consistent with the known microbiological coverage of vancomycin. Vancomycin has exclusive activity against Gram-positive organisms, which remain the predominant pathogens in many orthopaedic and spinal infections. Local administration enables delivery of supratherapeutic concentrations at the surgical site—levels that are difficult to achieve with systemic antibiotics, particularly in compromised or poorly perfused tissues—thereby potentially inhibiting early bacterial colonization^4^. This mechanism is particularly relevant in instrumented procedures, where implanted devices provide a substrate for bacterial adhesion and biofilm formation, thereby increasing the risk of persistent and treatment-resistant infection^34^. However, this effect does not extend to Gram-negative organisms, which likely explains the absence of a broader SSI signal.

Importantly, the lack of a consistent effect on deep or superficial SSI despite a clear organism-specific signal suggests that anatomical classifications of infection may be insufficiently sensitive to detect targeted antimicrobial effects. Deep infections are multifactorial events influenced by surgical technique, tissue viability, host immunity, and implant characteristics, in addition to microbial burden^33^. As such, even a biologically meaningful reduction in susceptible organisms may not translate into a measurable reduction in anatomically defined outcomes across heterogeneous surgical populations. This interpretation is supported by the VANCO trial^4^, in which the overall deep SSI endpoint narrowly missed statistical significance in time-to-event analysis despite a clear and statistically significant reduction in Gram-positive infections.

These findings have direct implications for clinical practice. SSI prevention is inherently multifactorial and already depends on the coordinated implementation of established evidence-based measures, including appropriate systemic antibiotic prophylaxis, skin preparation, perioperative glucose control, maintenance of normothermia, and related care bundles^1,2,5^. Contemporary antimicrobial stewardship frameworks further emphasize that progressively broader or more prolonged antibiotic strategies are unlikely to represent sustainable solutions to persistent SSI rates^3,35,36^. In this context, intrawound vancomycin should be regarded as a potential adjunct to, rather than a substitute for, established prevention pathways. In parallel, intraoperative wound irrigation represents another adjunctive strategy with variable supporting evidence. Recent randomized evidence suggests that antiseptic irrigation (such as that with povidone–iodine or chlorhexidine) may reduce SSI risk, whereas antibiotic irrigation is supported by evidence of lower certainty and raises similar antimicrobial stewardship concerns, reinforcing the preference for non-antibiotic adjuncts where feasible^37^.

The presence of vancomycin-resistant organisms, including vancomycin-resistant enterococci^38,39^, further underscores the importance of stewardship, as selective antimicrobial pressure may favour the emergence of predominance of pathogens not covered by vancomycin. Although no increase in Gram-negative SSI was observed, the confidence intervals remain compatible with smaller microbiological shifts, and local selective pressure at the wound interface may plausibly influence early colonization dynamics without necessarily translating into detectable changes in Gram-negative infection rates.

The data do not support routine or indiscriminate use of intrawound vancomycin across all surgical settings. Two prespecified findings support this context-specific interpretation directly. First, the relative benefit of intrawound vancomycin increased as control-group (baseline) SSI risk rose (meta-regression β = −0.0559 (s.e. 0.023); *P* = 0.017), indicating that the benefit is greatest where the underlying infection burden is highest. Second, the benefit was confined to Gram-positive SSI (RR 0.53), with no association for Gram-negative infection (RR 1.02), tying the expected benefit to the local microbiological ecology rather than to surgery *per se*. Rather, its use appears most justified in contexts where Gram-positive organisms predominate, local tissue conditions may limit systemic antibiotic penetration, and the consequences of infection are severe, such as in instrumented or implant-based procedures. Conversely, in low-risk procedures or settings with a higher prevalence of Gram-negative or polymicrobial infections, the expected benefit is likely negligible. This reinforces a risk-stratified approach to local antibiotic prophylaxis, rather than universal adoption.

The absence of any detectable effect on Gram-negative infection also highlights a key limitation of single-agent local antibiotic strategies. Combination approaches incorporating agents with complementary antimicrobial spectra (such as the addition of an aminoglycoside) may offer broader coverage and represent a rational direction for future investigation. Preclinical and early clinical studies suggest that such strategies may reduce polymicrobial infection risk, although robust randomized evidence remains limited^40,41^. Sensitivity analyses demonstrated that the effect estimate for Gram-positive SSI was robust to the exclusion of individual studies, whereas the estimate for overall SSI showed some dependence on influential trials. TSA indicated sufficient cumulative evidence for overall and Gram-positive SSI, with less certainty for deep and superficial SSI.

Taken together, these findings support a biologically targeted and context-specific role for intrawound vancomycin, rather than a universal strategy for SSI prevention.

Several limitations should be considered. First, although restricted to randomized trials, there remained variability in SSI definitions, follow-up duration, and procedural context, which may have attenuated effect estimates for anatomical outcomes. Second, most trials were not powered for organism-specific endpoints, and pathogen stratification was reported inconsistently, raising the possibility of outcome misclassification. Third, the included studies span diverse surgical specialties; although this enhances generalizability, it introduces clinical heterogeneity that may obscure procedure-specific effects. Although prespecified subgroup analyses by specialty, surgical site, and instrumentation status did not demonstrate significant effect modification, these analyses contained few trials per stratum, were underpowered, and cannot exclude procedure-specific differences; the pooled estimate should therefore be read as an average across procedures rather than as direct evidence of a uniform within-procedure effect. Finally, the modest absolute effect sizes raise the possibility that unmeasured confounding at the trial level (such as co-interventions or perioperative protocols) may have influenced observed outcomes despite randomization.

In this meta-analysis of randomized trials, intrawound vancomycin was associated with a modest reduction in SSI, primarily through a decrease in Gram-positive infections. These findings support a selective, context-specific role for intrawound vancomycin, particularly in higher-risk operative settings with a meaningful burden of Gram-positive infection, consistent with the organism-specific reduction in Gram-positive SSI and the greater relative benefit observed as baseline infection risk increased. Routine use across all procedures is not supported. Further patient-level analyses are needed to refine selection criteria and define how operative context and baseline risk influence treatment benefit, because aggregate trial-level data can identify effect modifiers but cannot establish the patient-level treatment thresholds required for individualized selection.

## Supplementary Material

zrag131_Supplementary_Data

## Data Availability

All data analysed in this study were extracted from previously published randomized clinical trials, which are cited in the reference list. The extracted dataset and analysis code are available from the corresponding author on reasonable request.
