# Supplementary material for: Intrawound vancomycin for prevention of surgical site infection: meta-analysis of randomized clinical trials
Source: BJS Open. 2026 Sep 21;10(5):zrag131. doi: 10.1093/bjsopen/zrag131 (PMC13591277; doi:10.1093/bjsopen/zrag131)
Supplement: Supplementary file 1 [file zrag131_supplementary_data.zip › Supplementary_Material.docx]

**Intrawound vancomycin for prevention of surgical site infection: meta-analysis of randomized controlled trials**

Shaan Patel MB BChir MA^1^†, Shiva A. Nischal MB BChir MA†^2^*, Kush M. Kale BA^2^, Angelette Mendonca BA^3^, Nelson Sofoluke MD^4^, Srinivas K. Prasad MD^1^, James S. Harrop MD^1^, Daniel Refai MD MBA^4^

^1^Department of Neurological Surgery, Thomas Jefferson University Hospital, Philadelphia, PA, USA

^2^Department of Physiology, Anatomy & Genetics, Medical Sciences Division, University of Oxford, Oxford, UK

^3^School of Clinical Medicine, University of Cambridge, Cambridge, UK

^4^Department of Neurological and Orthopedic Surgery, Emory University Hospital Midtown, Atlanta, GA, USA

†**These authors contributed equally to this work and share first authorship:**

Shaan Patel & Shiva A. Nischal

***Corresponding author:**

Shiva A Nischal, Department of Physiology, Anatomy & Genetics, Medical Sciences Division, University of Oxford, Sherrington Building, Sherrington Road, Oxford, OX1 3PT, UK ([shiva.nischal@sjc.ox.ac.uk](mailto:shiva.nischal@sjc.ox.ac.uk))

**Contents**

**Table S1.** Search strategies

**Table S2.** Summary of inclusion and exclusion criteria

**Table S3.** Funding status of included trials

**Table S4.** Baseline characteristics

**Table S5.** Interventions received

**Table S6.** Infection outcomes

**Table S7.** Postoperative outcomes

**Table S8.** Reported adverse events

**Table S9.** Sensitivity analyses for sparse-event primary outcomes

**Table S10.** Low risk of bias sensitivity analyses for primary outcomes

**Table S11.** Vancomycin powder-only sensitivity analyses for primary outcomes

**Table S12.** Meta-regression results

**Table S13.** Risk of bias assessment

**Table S14.** Summary of findings and certainty of evidence assessment

**Figure S1.** Forest plot for Gram-positive cocci

**Figure S2.** Forest plot for MRSA

**Figure S3.** Forest plot for Gram-negative SSI

**Figure S4.** Forest plot for reoperation

**Figure S5.** Forest plot for abscess

**Figure S6.** Forest plot for wound dehiscence

**Figure S7.** Forest plot for mortality

**Figure S8.** Forest plot for hospital length of stay

**Figure S9.** Leave-one-out analysis for overall SSI

**Figure S10.** Leave-one-out analysis for Gram-positive SSI

**Figure S11.** Leave-one-out analysis for Gram-positive cocci

**Figure S12.** Baujat plot for overall SSI

**Figure S13.** Baujat plot for Gram-positive SSI

**Figure S14.** Baujat plot for deep SSI

**Figure S15.** Baujat plot for superficial SSI

**Figure S16.** Subgroup analysis of overall SSI by vancomycin dose

**Figure S17.** Subgroup analysis of Gram-positive SSI by vancomycin dose

**Figure S18.** Subgroup analysis of deep SSI by vancomycin dose

**Figure S19.** Subgroup analysis of superficial SSI by vancomycin dose

**Figure S20.** Subgroup analysis of Gram-negative SSI by vancomycin dose

**Figure S21.** Subgroup analysis of reoperation by vancomycin dose

**Figure S22.** Subgroup analysis of abscess by vancomycin dose

**Figure S23.** Subgroup analysis of wound dehiscence by vancomycin dose

**Figure S24.** Subgroup analysis of mortality by vancomycin dose

**Figure S25.** Subgroup analysis of overall SSI by instrumentation status

**Figure S26.** Subgroup analysis of Gram-positive SSI by instrumentation status

**Figure S27.** Subgroup analysis of deep SSI by instrumentation status

**Figure S28.** Subgroup analysis of superficial SSI by instrumentation status

**Figure S29.** Subgroup analysis of Gram-negative SSI by instrumentation status

**Figure S30.** Subgroup analysis of overall SSI by surgical site

**Figure S31.** Subgroup analysis of Gram-positive SSI by surgical site

**Figure S32.** Subgroup analysis of deep SSI by surgical site

**Figure S33.** Subgroup analysis of superficial SSI by surgical site

**Figure S34.** Subgroup analysis of Gram-negative SSI by surgical site

**Figure S35.** Subgroup analysis of overall SSI by surgical specialty

**Figure S36.** Subgroup analysis of deep SSI by surgical specialty

**Figure S37.** Subgroup analysis of superficial SSI by surgical specialty

**Figure S38.** Subgroup analysis of overall SSI by SSI definition

**Figure S39.** Subgroup analysis of Gram-positive SSI by SSI definition

**Figure S40.** Subgroup analysis of deep SSI by SSI definition

**Figure S41.** Subgroup analysis of superficial SSI by SSI definition

**Figure S42.** Trial sequential analysis for overall SSI

**Figure S43.** Trial sequential analysis for Gram-positive SSI

**Figure S44.** Trial sequential analysis for deep SSI

**Figure S45.** Trial sequential analysis for superficial SSI

**Figure S46.** Meta-regression for overall SSI

**Figure S47.** Funnel plot for overall SSI

**Figure S48.** Funnel plot for Gram-positive SSI

**Figure S49.** Funnel plot for deep SSI

**Figure S50.** Funnel plot for superficial SSI

**Abbreviations**

IQR, interquartile range

MD, mean difference

n, number of patients

MRSA, methicillin-resistant *Staphylococcus aureus*

NR, not reported

RR, risk ratio
SD, standard deviation

SSI, surgical site infection

Vanc, vancomycin

**Table S1.** Search strategies. Full electronic search strategies used to identify relevant studies for this review, reported for each database. n = number of articles returned.

| **Database** | **Search Strategy** |
| --- | --- |
| Medline  (n=595) | "Vancomycin"[MeSH Terms] OR "vancomycin*"[tiab] OR "vancocin*"[tiab]  AND  "Administration, Topical"[MeSH Terms] OR "topical*"[tiab] OR "intrawound*"[tiab] OR "intra-wound*"[tiab] OR "powder*"[tiab] OR "local*"[tiab] OR "intraoperative*"[tiab] OR "paste*"[tiab] OR "carrier*"[tiab] OR "prophylaxis"[tiab]  AND  "randomized controlled trial"[pt] OR "controlled clinical trial"[pt] OR “randomized"[tiab] OR "placebo"[tiab] OR "clinical trials as topic"[MeSH] OR "randomly"[tiab] OR "trial"[tiab] OR "groups"[tiab] |
| Embase  (n=3171) | 'vancomycin'/exp OR vancomycin*:ti,ab OR vancocin*:ti,ab  AND  'topical drug administration'/exp OR 'topical application'/exp OR  (topical* OR intrawound* OR 'intra wound' OR powder* OR local* OR intraoperat* OR paste* OR prophylaxis):ti,ab  AND  'randomized controlled trial'/exp OR 'randomization'/exp OR 'double-blind procedure'/exp OR 'single-blind procedure'/exp OR (random* OR factorial* OR crossover* OR "cross over" OR placebo* OR "double blind" OR "single blind" OR assign*):ti,ab |
| CENTRAL  (n=324) | [mh Vancomycin] OR (vancomycin* OR vancocin*):ti,ab,kw  AND  [mh "Administration, Topical"] OR (topical* OR intrawound* OR "intra-wound" OR powder* OR local* OR intraoperat* OR intrasurg* OR prophylaxis OR carrier* OR paste* OR "direct application*" OR "intraoperative application*"):ti,ab,kw |

**Table S2.** Summary of inclusion and exclusion criteria. GCS, Glasgow Coma Scale; ORIF, open reduction and internal fixation.

| **Author (year)** | **Inclusion criteria** | **Exclusion criteria** |
| --- | --- | --- |
| Abuzaiter (2023) | Aged 18–90 years; referred for total knee arthroplasty | Vancomycin allergy; diagnosis of active cancer; systemic instability (sepsis, hemodynamic instability, signs of active infection); previous surgery; history of radiation therapy at the surgical site |
| Acosta-Olivo (2024) | Age >18 years; closed fracture (any mechanism/classification) requiring ORIF | Open fractures; immunodeficiency or immunosuppressant/steroid use; vancomycin allergy; ankle surgery <6 months prior; pregnancy; antibiotic therapy <1 week prior to admission. |
| Atallah (2021) | Age 16–65 years; head trauma; GCS 9–15 on admission; fit for surgery. | Age <16 or >65 years; elective cranial surgery; high-risk medical condition; GCS <9 |
| do Nascimento (2019) | Age >16 years; thoracolumbar spine fracture or degenerative disease; indicated for spinal arthrodesis | Prior spinal surgery; prior spinal infection; pathological fractures due to tumors; refusal to participate |
| Gandhi (2023) | Isolated closed fractures; planned for ORIF within <2 weeks from injury | Immunosuppressive therapy; prior surgical site infection; vancomycin allergy; pathological or peri-prosthetic fractures; polytrauma; remote infection foci; platelet count <150,000/mL; total lymphocyte count <1500/mm^3^; metabolic bone disease |
| Han (2024) | Severe symptoms from degenerative lumbar spine disorders; lumbar instability or Pfirrmann grade >III disc degeneration; failed conservative treatment; informed consent; minimum follow-up >90 days | Hepatic or renal impairment; cephalosporin or vancomycin allergy; recent glucocorticoid use; local or systemic infection; psychological disorders, alcoholism, or substance abuse; pregnancy; hearing impairment |
| Hasan (2020) | Disc prolapse; failure of conservative treatment; surgical indication | Prior open discectomy (revision surgery); vancomycin allergy; requirement for spinal instrumentation |
| Kannan (2025) | Age ≥18 years; craniotomy with planned dural/dural substitute closure OR non-instrumented spinal procedure | Carotid endarterectomy; transsphenoidal procedures; vestibular schwannoma diagnosis; instrumented spinal procedures; renal insufficiency; vancomycin allergy |
| Khalid (2023) | Age 16–65 years; traumatic thoracolumbar fracture, spinal metastasis, spinal tuberculosis, or degenerative spinal disease; planned for posterior spinal instrumentation | Prior spinal instrumentation surgery (clean or infected) |
| Kunakornsawat (2019) | Instrumented posterior thoracic or lumbosacral spine surgery | Open injuries; history of surgical site infection; current infection; follow-up <3 months; vancomycin allergy; refusal of consent. |
| Mirzashahi (2017) | Patients undergoing open spine surgery | Infection <1 month; current antibiotic use; pregnancy; history of Stevens-Johnson syndrome; prior surgical site infection; immunosuppressive drugs or immunodeficiency disease; prior radiation at surgical site; surgery for spondylodiscitis; urinary incontinence |
| Mohammadhoseini (2024) | Age >18 years; candidates for primary hip hemiarthroplasty; informed consent | Active local or systemic infection; osteoarthritis caused by infection; revision surgery; vancomycin allergy |
| Morgan (2024) | Age ≥18 years; undergoing clean cranial surgery (craniotomy, craniectomy, autologous cranioplasty) or clean spine surgery (laminectomy, discectomy, laminoplasty) | Pre-existing infection at or adjacent to surgical site; presence of cranial or spinal implants |
| Mulpur (2024) | Adult patients; primary knee osteoarthritis; planned for primary manual jig-based total knee arthroplasty; informed consent | Refusal to participate; intraoperative findings of inflammatory arthropathy or nonspecific synovitis; prior knee surgery; intra-articular injections <3 years; vancomycin allergy; chronic immunosuppression; antibiotic use <1 month till surgery |
| O’Toole (2021) | Age 18–80 years; tibial plateau or pilon fracture requiring plate/screw fixation and deemed "high risk" (defined as: definitive fixation >3 days post-injury due to swelling/external fixation; Gustilo-Anderson Type I, II, or IIIA open fracture; or ipsilateral leg compartment syndrome requiring fasciotomy) | Infection at enrollment; Gustilo-Anderson Type IIIB or IIIC open fracture; vancomycin allergy |
| Rathore (2025) | Age >18 years; open long bone fractures (Gustilo-Anderson Type I, II, IIIA, or IIIB) | Age <18 years; type 2 diabetes mellitus; HIV infection or other immunocompromise; Gustilo-Anderson Type IIIC fractures; refusal of consent |
| Saba (2025) | Age >18 years; primary elective unilateral total joint arthroplasty and ≥ 1 "high-risk" criterion: age >75, BMI >40, active smoker, ASA ≥3, immunosuppression, *S. aureus* colonization | Active pregnancy; inability to consent; severe dementia; active infection or wound of operative limb; vancomycin or iodine allergy; prior native septic arthritis; ASA score 5 or 6; planned procedure <90 days post-surgery |
| Salimi (2021) | Age >18 years; undergoing open spinal surgery (cervical, thoracic, or lumbosacral) | Prior spine infection; immunodeficiency or ongoing chemotherapy; renal or hepatic failure; vancomycin or cephalosporin allergy; follow-up <3 months; primary or metastatic spinal/spinal cord tumors; refusal of consent |
| Singh (2019) | Age 20–65 years; mandibular fracture requiring ORIF; informed consent | Prior infection at surgical site; vancomycin allergy; pathological fracture; systemic disease or immunocompromised status; follow-up <6 months |
| Tubaki (2013) | Patients undergoing open spine surgery at any level | Prior infection at surgical site; biopsy procedure; follow-up <12 weeks; vancomycin allergy; minimally invasive spine surgery |
| Wang (2025) | Degenerative lumbar disease; failed conservative treatment 3–6 months; undergoing both decompression and instrumented fusion | Decompression-only procedure; minimally invasive surgery; active malignancy or osteomyelitis; vertebral fracture; refusal of consent; dynamic fixation; death or loss to follow-up post-discharge |
| Yusuf (2026) | Patients undergoing cranial surgery | Open or penetrating cranial injuries; contaminated or dirty wounds; reoperation for surgical site infection debridement; vancomycin allergy |

**Table S3.** Funding status of included trials.

| **Author (year)** | **Funding statement** |
| --- | --- |
| Abuzaiter (2023) | This research did not receive any specific grant from funding agencies in the public, commercial, or not-for-profit sectors. |
| Acosta-Olivo (2024) | All authors have declared that no financial support was received from any organization for the submitted work |
| Atallah (2021) | Nil financial support or sponsorship |
| do Nascimento (2019) | The authors report no conflict of interest concerning the materials or methods used in this study or the findings specified in this paper. |
| Gandhi (2023) | Nil funding |
| Han (2024) | No applicable funding |
| Hasan (2020) | NR |
| Kannan (2025) | Funding for this work was provided by the National Institutes of Health (NIH) through the Agency of Healthcare Research and Quality (AHRQ). |
| Khalid (2023) | NR |
| Kunakornsawat (2019) | Nil financial support or sponsorship |
| Mirzashahi (2017) | The authors declare that they have no conflict of interest |
| Mohammadhoseini (2024) | The authors declare that they have no competing interests |
| Morgan (2024) | No funding was received for conducting this study |
| Mulpur (2024) | No financial conflicts of interest to declare |
| O’Toole (2021) | The study was funded by grant W81XWH-10-2-0134 from the DOD’s Congressionally Directed Medical Research Program (Drs O’Toole, Castillo, and Carlini). |
| Rathore (2025) | NR |
| Saba (2025) | One or more of the authors of this paper have disclosed potential or pertinent conflicts of interest, which may include receipt of payment, either direct or indirect, institutional support, or association with an entity in the biomedical field which may be perceived to have potential conflict of interest with this work |
| Salimi (2021) | The study received no funding |
| Singh (2019) | Nil financial support or sponsorship |
| Tubaki (2013) | No relevant financial activities outside the submitted work |
| Wang (2025) | The authors declare that they have no known competing financial interests or personal relationships that could have appeared to influence the work reported in this paper. |
| Yusuf (2026) | Nil financial support or sponsorship |

**Table S4.** Baseline characteristics. Inst., instrumented; THA, total hip arthroplasty; TKA, total knee arthroplasty; Uninst., uninstrumented.

| **Author (year)** | **Country** | **Age, mean ± SD or median (IQR), y** | | **Male sex, %** | | **BMI, mean ± SD or median (IQR), kg/m^2^** | | **Type 2 diabetes mellitus, n/N** | | **Smokers, n/N** | | **Hypertension, n/N** | | **Immunosuppressed, n/N** | |
| --- | --- | --- | --- | --- | --- | --- | --- | --- | --- | --- | --- | --- | --- | --- | --- |
|  |  | **Vanc** | **Control** | **Vanc** | **Control** | **Vanc** | **Control** | **Vanc** | **Control** | **Vanc** | **Control** | **Vanc** | **Control** | **Vanc** | **Control** |
| Abuzaiter (2023) | Canada | 66 | 64 | 41.3 | 34.1 | 33.4 | 35.7 | 16/80 | 13/85 | 9/80 | 4/85 | NR | NR | NR | NR |
| Acosta-Olivo (2024) | Mexico | 37.7 ± 14.6 | 40.1 ± 13.0 | 49.2 | 48.5 | NR | NR | 4/66 | 6/66 | NR | NR | 6/66 | 0/66 | NR | NR |
| Atallah (2021) | Egypt | 32.1 ± 15.9 | 31.9 ± 16.7 | 87.2 | 83 | NR | NR | 1/39 | 2/47 | 21/39 | 25/47 | 4/39 | 6/47 | NR | NR |
| do Nascimento (2019) | Italy | 43.0 ± 14.9 | 45.0 ± 15.0 | 81.6 | 66.0 | NR | NR | 5/49 | 5/47 | 8/49 | 6/47 | 7/49 | 7/47 | NR | NR |
| Gandhi (2023) | India | 40.4 ± 14.2 | 40.9 ± 15.6 | 81.8 | 65.9 | NR | NR | 1/44 | 4/44 | NR | NR | 1/44 | 1/44 | 0/44 | 0/44 |
| Han (2024) | China | 59.3 ± 13.3 | 62.9 ± 8.6 | 44.0 | 45.0 | 26.5 ± 4.2 | 25.4 ± 4.3 | 16/78 | 21/78 | 4/78 | 11/78 | 39/78 | 47/78 | NR | NR |
| Hasan (2020) | Iraq | 38.2 ± 8.1 | 34.7 ± 7.2 | 91.2 | 69.2 | NR | NR | 9/192 | 7/198 | 39/192 | 33/198 | NR | NR | 3/192 | 1/198 |
| Kannan (2025) | USA | 54.2 ± 16.0 | 53.9 ± 16.5 | 42.9 | 44.1 | 28.1 ± 11.8 | 27.6 ± 8.1 | 41/552 | 38/551 | 34/552 | 25/551 | 158/552 | 138/551 | 328/552 | 320/551 |
| Khalid (2023) | Pakistan | 36.0 ± 16.6 | 33.7 ± 15.9 | 53.8 | 58.9 | 22.1 ± 1.8 | 22.3 ± 1.5 | 4/39 | 2/39 | 1/39 | 1/39 | 3/39 | 2/39 | NR | NR |
| Kunakornsawat (2019) | Thailand | 2g: 53.2 ± 15.0  1g: 51.0 ± 14.0 | 55.2 ± 14.5 | 2g: 43.5  1g: 41.8 | 36.3 | 2g: 27.0  1g: 28.9 | 25.8 | 2g: 8/131  1g: 11/134 | 11/135 | 2g: 4/131  1g: 4/134 | 7/135 | 2g: 31/131  1g: 27/134 | 30/135 | NR | NR |
| Mirzashahi (2017) | Iran | 42.1 ± 20.1 | 61.4 ± 9.4 | 35.5 | | >30 (n=2) | >30 (n=3) | 12/193 | 10/187 | 18/193 | 14/187 | NR | NR | NR | NR |
| Mohammadhoseini (2024) | Iran | 72.0 ± 5.3 | 74.0 ± 3.5 | 41.6 | 41.6 | NR | NR | NR | NR | NR | NR | NR | NR | NR | NR |
| Morgan (2024) | Nigeria | 48.1 ± 17.1 | 46.0 ± 19.1 | 66.7 | 85.7 | 23.9 ± 5.2 | 23.2 ± 3.0 | 9/21 | 8/21 | 2/21 | 3/21 | NR | NR | 4/21 | 0/21 |
| Mulpur (2024) | India | 61.7 ± 7.5 | 61.4 ± 7.4 | 29 | 30.5 | 28.5 ± 4.5 | 28.4 ± 4.5 | 170/507 | 161/515 | 58/507 | 60/515 | NR | NR | NR | NR |
| O'Toole (2021) | USA | 45.4 ± 14.0 | 46.1 ± 13.5 | 62.2 | 63.7 | 29.9 ± 7.3 | 29.9 ± 7.0 | 45/481 | 48/499 | 190/481 | 193/499 | NR | NR | NR | NR |
| Rathore (2025) | India | 44.1 ± 13.4 | 43.2 ± 12.5 | 78.1 | 71.9 | NR | NR | 0/64 | 0/64 | 0/64 | 0/64 | NR | NR | 0/64 | 0/64 |
| Saba (2025) | USA | THA: 65.0 ± 12.5  TKA: 67.0 ± 10.8 | THA: 65.0 ± 12.5  TKA: 67.0 ± 10.8 | THA: 41.7  TKA: 30.5 | THA: 42.5  TKA: 32.3 | NR | NR | THA: 49/199 TKA: 79/256 | THA: 38/199 TKA: 91/256 | THA: 27/199 TKA: 19/256 | THA: 30/199 TKA: 24/256 | NR | NR | NR | NR |
| Salimi (2021) | Iran | 51.7 | 52.3 | 42.2 | 44.7 | >30 (n=23) | >30 (n=25) | 14/187 | 12/188 | 42/187 | 45/188 | 28/187 | 25/188 | NR | NR |
| Singh (2019) | India | 32.5 | 33.2 | 80.0 | 84.0 | NR | NR | 0/50 | 0/50 | 0/50 | 0/50 | 0/50 | 0/50 | 0/50 | 0/50 |
| Tubaki (2013) | India | Inst.: 44.5 ± 19.8  Uninst.: 43.7 ± 16.5 | Inst.: 46.6 ± 20.0  Uninst.: 46.7 ± 19.3 | Inst.: 49.0  Uninst.: 66 | Inst.: 50.6  Uninst.: 70.5 | NR | NR | Inst.: 52/302  Uninst.: 23/131 | Inst.: 52/304  Uninst.: 25/170 | NR | NR | Inst.: 68/302  Uninst.: 21/131 | Inst.: 49/302  Uninst.: 27/170 | NR | NR |
| Wang (2025) | Taiwan | 68.1 ± 11.3 | 67.3 ± 10.7 | 36.7 | 37.4 | 26.5 ± 4.2 | 26.9 ± 4.4 | 119/357 | 105/348 | 55/357 | 49/348 | NR | NR | NR | NR |
| Yusuf (2026) | Nigeria | 40.0 ± 12.8 | 42.0 ± 14.2 | 59.6 | 63.5 | NR | NR | 14/52 | 7/52 | 4/52 | 16/52 | 22/52 | 25/52 | 15/52 | 14/52 |

**Table S5.** Interventions received. CDC, Centers for Disease Control and Prevention; IV, intravenous; MSIS, Musculoskeletal Infection Society; ORIF, open reduction and internal fixation.

| **Author (year)** | **Vanc dose, g** | **Vanc method of application** | **Standard antibiotic prophylaxis (both groups)** | **Operative details** | | **Surgical site infection definition** | **Duration of follow-up, months** |  |
| --- | --- | --- | --- | --- | --- | --- | --- | --- |
|  |  |  |  | **Vanc** | **Control** |  |  |  |
| Abuzaiter (2023) | 1 | 0.5g powder applied directly to prosthesis, and 0.5g above the closed joint capsule. | 2–3g preoperative IV cefazolin | Total knee arthroplasty | | MSIS criteria | 1.5 |  |
| Acosta-Olivo (2024) | 1 | Powder applied directly to surgical wound after fixation. | 1g preoperative IV cefalotin, followed by IV cefalotin every 8 hours for the next 24 hours during hospital stay. | ORIF for ankle fracture:  Weber type B (n=43)  Weber type C (n=16)  Medial malleolar (n=6) | ORIF for ankle fracture:  Weber type B (n=49)  Weber type C (n=10)  Medial malleolar (n=5) | CDC criteria | 3 |  |
| Atallah (2021) | 1 | Powder applied in the subgaleal space. | IV cefazolin within 30 mins of incision, continued for 48 hours | Unilateral extradural hematoma (n=8)  Bilateral extradural hematoma (n=1)  Traumatic intracerebral hematoma (n=2)  Compound depressed fractures (n=23)  Simple depressed fractures (n=1)  Acute subdural hematoma (n=3)  Penetrating brain injury (n=1) | Unilateral extradural hematoma (n=19)  Bilateral extradural hematoma (n=2)  Traumatic intracerebral hematoma (n=2)  Compound depressed fractures (n=19)  Simple depressed fractures (n=3)  Acute subdural hematoma (n=1)  Penetrating brain injury (n=1) | Culture-positive wound infection within 120d | 4 |  |
| do Nascimento (2019) | 2 | Vancomycin paste (2g in 20mL saline) applied at the surgical site before closing the aponeurosis. | Standard antibiotic prophylaxis (non-specified) | Thoracolumbar fusion:  T1–T11 (n=22)  T12–L1 (n=17)  L2 and below (n =10) | Thoracolumbar fusion:  T1–T11 (n=14)  T12–L1 (n=18)  L2 and below (n=15) | CDC criteria | 5 |  |
| Gandhi (2023) | 2 | Powder applied to the fracture site (on muscle, fascia, and subcutaneous tissue), after wound wash, and before closure. | 1g IV ceftriaxone at incision, followed by 1g IV ceftriaxone every 8 hours for 24 hours | ORIF for closed fractures:  Upper limb (n=21)  Lower limb (n=16)  Spine injury (n=6)  Pelvic injury (n=1)  Multiple fractures (n=8) | ORIF for closed fractures:  Upper limb (n=14)  Lower limb (n=20)  Spine injury (n=5)  Pelvic injury (n=5)  Multiple fractures (n=10) | International consensus definition of fracture-related infection | 1.5 |  |
| Han (2024) | 1 | Powder applied over the muscle, fascia, and subcutaneous layers before skin closure. | 1.5g IV cefuroxime within 1 hour of incision (dose repeated if surgery lasted >3 hours) | Posterior lumbar interbody fusion:  Lumbar spinal stenosis (n=54)  Lumbar spondylolisthesis (n=15)  Lumbar disc herniation (n=9) | Posterior lumbar interbody fusion:  Lumbar spinal stenosis (n=46)  Lumbar spondylolisthesis (n=18)  Lumbar disc herniation (n=14) | CDC criteria | 3 |  |
| Hasan (2020) | 1–2 | Powder inserted into the disc space using a bone graft funnel after microscopic tubular discectomy. | 1g IV cefazolin 1 hour before surgery, then 2 postoperative doses of 1g cefazolin IV, 12 hours apart | Microscopic tubular discectomy:  L1–2 (n=0)  L2–3 (n=1)  L3–4 (n=6)  L4–5 (n=90)  L5–S1 (n=95) | Microscopic tubular discectomy:  L1–2 (n=1)  L2–3 (n=1)  L3–4 (n=4)  L4–5 (n=101)  L5–S1 (n=91) | Postoperative discitis (clinical diagnosis) | 3 |  |
| Kannan (2025) | 2 | 1g was kept as powder and 1g was mixed with saline to form a paste, which was applied to the edges and outer surface of the craniotomy/surgical site prior to closure of the skin or aponeurosis. | IV cefazolin 1 hour prior to incision | Craniotomy (n=338)  Craniectomy (n=71)  Cranioplasty (n=7)  Spine (n=12)  Other (n=27) | Craniotomy (n=340)  Craniectomy (n=55)  Cranioplasty (n=14)  Spine (n=17)  Other (n=39) | Readmission or positive wound culture | 1 |  |
| Khalid (2023) | 1 | Powder applied over the implant and in the deep subcutaneous tissue prior to wound closure. | 2g preoperative IV cefoperazone + sulbactam within 30 minutes of incision. Postoperative 1g IV cefoperazone + sulbactam every 12 hours for 3 days | Instrumented posterior spine surgery:  Spinal metastases (n=1)  Traumatic fracture (n=28)  Degenerative disease (n=4)  Spine tuberculosis (n=6) | Instrumented posterior spine surgery:  Spinal metastases (n=0)  Traumatic fracture (n=35)  Degenerative disease (n=1)  Spine tuberculosis (n=3) | Infection occurring within 3 months post-surgery requiring additional operation and antibiotic treatment | 3 |  |
| Kunakornsawat (2019) | 1–2 | Powder spread throughout the surgical wound before skin closure (half mixed with bone graft; half spread in the wound). | 1g preoperative IV cefazolin within 60 min of incision  Postoperatively switched to oral antibiotics (dicloxacillin or clindamycin) after drain removal for 7 days. | Instrumented posterior spine surgery | | CDC criteria | 3 |  |
| Mirzashahi (2017) | 1–2 | Powder applied locally over the fascia after fascial closure but before final skin closure. | 1g or 2g (weight-dependent) preoperative IV cefazolin 20 min before incision, repeated every 3 hours during surgery or earlier if blood loss exceeded 1000mL. | Single level open posterior spine surgery:  Degenerative pathology and tumors (n=304)  Trauma (n=42)  Spine deformity (n=34) | | CDC criteria | 3 |  |
| Mohammadhoseini (2024) | 1 | Intra-articular powder suspension administered during the operation before closure of the fascia | 2g IV cefazolin administered 30 minutes before skin incision | Hip hemiarthroplasty | | MSIS criteria | 6 |  |
| Morgan (2024) | 1 | Powder poured into the wound cavity (subfascial and subcutaneous plane) before closure. | 1g IV ceftriaxone at anesthetic induction | Cranial (n=12)  Spine (n=9) | Cranial (n=20)  Spine (n=1) | CDC criteria | 1 |  |
| Mulpur (2024) | 2 | Powder applied into the medial and lateral joint gutters before arthrotomy closure (after tourniquet deflation and hemostasis). | 3 perioperative IV doses of 1.5g cefuroxime | Total knee arthroplasty | | MSIS criteria | 12 |  |
| O’Toole (2021) | 1 | Sterile powder placed directly over metal implants in the surgical wound during definitive fracture fixation. | Systemic IV antibiotics at definitive fixation (cefazolin, vancomycin, clindamycin, gentamicin, cephalexin, trimethoprim and sulfamethoxazole, levofloxacin, ceftriaxone, or cefepime) | High-risk tibia fractures:  Gustilo-Anderson I (n=8)  Gustilo-Anderson II (n=28)  Gustilo-Anderson IIIA (n=63)  Closed fracture (n=381)  NR (n=1) | High-risk tibia fractures:  Gustilo-Anderson I (n=10)  Gustilo-Anderson II (n=30)  Gustilo-Anderson IIIA (n=51)  Closed fracture (n=408)  NR (n=0) | CDC criteria | 6 |  |
| Rathore (2025) | 1 | Sterile powder placed directly over metal implants in the surgical wound at the time of definitive fracture fixation. | Standard antibiotic prophylaxis (non-specified) | Fixation of open long bone fracture:  Gustilo-Anderson I (n=15)  Gustilo-Anderson II (n=15)  Gustilo-Anderson IIIA (n=18)  Gustilo-Anderson IIIB (n=16) | Fixation of open long bone fracture:  Gustilo-Anderson I (n=13)  Gustilo-Anderson II (n=25)  Gustilo-Anderson IIIA (n=13)  Gustilo-Anderson IIIB (n=13) | Infection within 30d of surgery (or 1 yr if implant) | 6 |  |
| Saba (2025) | 2 | Powder distributed as 1g deep to the fascia and 1g superficial to the fascia. | Standard antibiotic prophylaxis (non-specified) | Total hip arthroplasty (n=199)  Total knee arthroplasty (n=256) | Total hip arthroplasty (n=187)  Total knee arthroplasty (n=297) | International Consensus Meeting criteria for periprosthetic joint infection | 3 |  |
| Salimi (2021) | 1–2 | Powder applied to muscles, bone, and subcutaneous tissue before closure (but specifically not to the dura or fusion site). | 2g preoperative IV cephalosporin (<2 hours before surgery), repeated if surgery lasted >2 hours. Postoperatively, 1g IV cephalosporin every 8 hours for 24 hours | Spine surgery:  Instrumented (n=72)  Non-instrumented (n=115) | Spine surgery:  Instrumented (n=75)  Non-instrumented (n=113) | CDC criteria | 3 |  |
| Singh (2019) | 1 | Powder applied liberally over hardware and wound prior to closure | Standard antibiotic prophylaxis (non-specified) | ORIF of mandibular fracture | | Clinical parameters, including postoperative fever, abnormal swelling, any purulent discharge, and fistula formation. | 12 |  |

| Tubaki (2013) | 1 | Powder spread throughout the surgical wound before closure (on muscle, fascia, and subcutaneous tissue, but explicitly avoiding bone graft and dura). | | 750mg IV cefuroxime just before incision followed by 750mg IV cefuroxime every 8 hours for 1 day (non-instrumented spine procedures) or until drain removal (instrumented). | Instrumented spine surgery:  Lumbar (n=195)  Thoracolumbar (n=35)  Cervical (n=37)  Thoracic (n=35)  Uninstrumented spine surgery:  Lumbar (n=118)  Cervical (n=10)  Thoracic (n=2)  Occipital (n=1) | Instrumented spine surgery:  Lumbar (n=201)  Thoracolumbar (n=25)  Cervical (n=44)  Thoracic (n=34)  Uninstrumented spine surgery:  Lumbar (n=138)  Cervical (n=20)  Thoracic (n=9)  Occipital (n=2)  Coccyx (n=1) | Clinical evidence of infection | 3 |
| --- | --- | --- | --- | --- | --- | --- | --- | --- |
| Wang (2025) | 1–2 | Powder mixed homogeneously with a 1:1 mixture of bone graft (autogenous) and bone substitute (β-tricalcium phosphate). Mixture was left undisturbed for 30 minutes before application to allow adherence. | 1g IV cephalosporin 30 min before surgery, repeated every 4 hours or if blood loss >1000mL. 1g postoperative IV cephalosporin every 8h plus 80mg aminoglycoside every 12h for 3 days. | | Elective lumbar instrumented fusion surgery:  2–3 levels (n=300)  >3 levels (n=57) | Elective lumbar instrumented fusion surgery:  2–3 levels (n=291)  >3 levels (n=57) | CDC criteria | 12 |
| Yusuf (2026) | 1 | Powder distributed evenly in subgaleal and supragaleal layers prior to wound closure. | IV ceftriaxone preoperatively | | Major cranial surgery | | CDC criteria | 3 |

**Table S6.** Infection outcomes

| **Author (year)** | **Surgical site infection, n/N** | | **Superficial surgical site infection, n/N** | | **Deep surgical site infection, n/N** | | **Periprosthetic joint infection, n/N** | | **Bacteria cultured at surgical site (n)** | | **Time to SSI, days** | | |
| --- | --- | --- | --- | --- | --- | --- | --- | --- | --- | --- | --- | --- | --- |
|  | **Vanc** | **Control** | **Vanc** | **Control** | **Vanc** | **Control** | **Vanc** | **Control** | **Vanc** | **Control** | **Vanc** | **Control** |  |
| Abuzaiter (2023) | 3/80 | 0/85 | NR | NR | 3/80 | 0/85 | 3/80 | 0/85 | *E. cloacae* (1)  *P. mirabilis* (1)  Coagulase-negative staphylococcus (1) | NR | NR | NR |  |
| Acosta-Olivo (2024) | 2/66 | 3/66 | NR | NR | 2/66 | 3/66 | NR | NR | *S. aureus* (1)  *A. baumannii* (1) | *S. aureus* (1) | <14d (n=2) | <14d (n=2)  14–28d (n=1) |  |
| Atallah (2021) | 1/39 | 7/47 | NR | NR | 1/39 | 7/47 | NR | NR | *Acinetobacter* spp. (1) | *Acinetobacter* spp. (1)  MRSA (3)  MRSA and *Klebsiella* (1)  No growth (2). | 15 | 15 ± 3.2 |  |
| do Nascimento (2019) | 4/49 | 4/47 | NR | NR | 4/49 | 4/47 | NR | NR | MSSA (2)  *E. cloacae* (1)  *P. aeruginosa* (1) | MSSA (1)  *Acinetobacter* spp. (1)  *C. freundii* (1)  *P. stuartii* (1)  *E. cloacae* (1) | NR | NR |  |
| Gandhi (2023) | 0/44 | 2/44 | 0/44 | 2/44 | 0/44 | 0/44 | NR | NR | NR | *P. aeruginosa* (n=1)  *A. baumannii* (n=1) | N/A | <42d |  |
| Han (2024) | 1/78 | 8/78 | 1/78 | 7/78 | 0/78 | 1/78 | NR | NR | *P. aeruginosa* (1) | *S. aureus* (2)  *E. coli* (2)  *Klebsiella* spp. (1)  *S. pneumoniae* (1)  Unidentified (2) | <90d | |  |
| Hasan (2020) | 2/192 | 11/198 | NR | NR | 2/192 | 11/198 | NR | NR | *S. aureus* (1)  *E. coli* (1) | *S. aureus* (2)  *S. epidermidis* (1)  Gram-negative bacilli (1) | 9.0 ± 1.0 | 14.5 ± 6.0 |  |
| Kannan (2025) | 23/552 | 23/551 | 18/552 | 19/551 | 5/552 | 4/551 | NR | NR | NR | NR | 14–30d | |  |
| Khalid (2023) | 1/39 | 5/39 | 1/39 | 2/39 | 0/39 | 3/39 | NR | NR | *E. coli* (1) | *S. aureus* (2)  *S. epidermidis* (1)  *P. aeruginosa* (1)  *E. coli* (1) | <90d | |  |
| Kunakornsawat (2019) | 1g: 4/134  2g: 5/131 | 4/135 | NR | NR | 1g: 5/134  2g: 4/131 | 4/135 | NR | NR | 2g: *P. aeruginosa* (1) *Staphylococcus* spp. (1) Diphtheroids (1)  No growth (2)  1g: *S. aureus* (2)  No growth (2) | *Staphylococcus* spp. (1)  No growth (3) | 15 (4–31) | |  |
| Mirzashahi (2017) | 10/193 | 5/187 | NR | NR | 10/193 | 5/187 | NR | NR | *Acinetobacter* spp. (2)  *P. aeruginosa* (2) | *S. aureus* (2)  *Acinetobacter* spp. (2)  MRSA (1) | <90d | |  |
| Mohammadhoseini (2024) | 3/24 | 3/24 | 2/24 | 1/24 | 1/24 | 2/24 | 1/24 | 2/24 | NR | NR | <180d | |  |
| Morgan (2024) | 0/21 | 7/21 | 0/21 | 7/21 | 0/21 | 0/21 | NR | NR | NR | *S. aureus* (2)  *K. pneumoniae* (1)  *P. aeruginosa* (1)  No growth (3) | <30d | |  |
| Mulpur (2024) | 2/507 | 5/515 | 1/507 | 2/515 | 1/507 | 3/515 | 1/507 | 3/515 | *S. epidermidis* (1)  No growth (1) | *S. aureus* (1)  No growth (4) | <365d | |  |
| O’Toole (2021) | 46/481 | 60/499 | 17/481 | 14/499 | 29/481 | 46/499 | NR | NR | Gram-positive bacilli (16)  Gram-negative bacilli (11) | Gram-positive bacilli (34)  Gram-negative bacilli (10) | <180d | |  |
| Rathore (2025) | 4/64 | 5/64 | NR | NR | NR | NR | NR | NR | NR | NR | NR | NR |  |
| Saba (2025) | 3/455 | 2/484 | NR | NR | 3/455 | 2/484 | 3/455 | 2/484 | NR | NR | NR | NR |  |
| Salimi (2021) | 12/187 | 13/188 | 5/187 | 7/188 | 7/187 | 6/188 | NR | NR | Instrumented:  *Klebsiella* (2)  *S. aureus* (1)  *Pseudomonas* spp. (1)  No growth (1)  Non-instrumented:  *S. aureus* (2)  *S. epidermidis* (1) *Pseudomonas* spp. (1)  *Acinetobacter* spp. (1) *Klebsiella* spp. + *S. epidermidis* (1)  No growth (1) | Instrumented:  *S. aureus* (2)  *S. epidermidis* (2)  *Klebsiella* (1)  Non-instrumented  *S. aureus* (4)  *S. epidermidis* (2)  *E. coli* (1)  No growth (1) | NR | NR |  |
| Singh (2019) | 2/50 | 8/50 | NR | NR | NR | NR | NR | NR | *Staphylococcus* spp. + MRSA (2) | *Staphylococcus* spp. + MRSA (8) | NR | NR |  |
| Tubaki (2013) | 7/433 | 8/474 | 1/433 | 2/474 | 6/433 | 6/474 | NR | NR | *S. aureus* (1)  *Klebsiella* spp. (2)  No growth (4) | *S. aureus* (2)  *E. coli* (1)  No growth (5) | NR | NR |  |
| Wang (2025) | 7/357 | 8/348 | 7/357 | 3/348 | 0/357 | 5/348 | NR | NR | NR | MRSA (3) | NR | NR |  |
| Yusuf (2026) | 5/52 | 6/52 | NR | NR | NR | NR | NR | NR | *S. aureus* (4)  *E. cloacae* (1) | *S. aureus* (6) | <30d | |  |

**Table S7.** Postoperative outcomes. Inst., instrumented; THA, total hip arthroplasty; TKA, total knee arthroplasty; Uninst., uninstrumented.

| **Author (year)** | **Operative time, minutes** | | **Length of stay, mean ± SD, days** | | **Patients requiring blood transfusion, n/N** | | **Intraoperative blood loss, mean ± SD, mL** | | **Reoperation, n/N** | |
| --- | --- | --- | --- | --- | --- | --- | --- | --- | --- | --- |
|  | **Vanc** | **Control** | **Vanc** | **Control** | **Vanc** | **Control** | **Vanc** | **Control** | **Vanc** | **Control** |
| Abuzaiter (2023) | NR | NR | >5 (n=9) | >5 (n=10) | 1/80 | 5/85 | <30ml (n=43) >30ml (n=22) | <30ml (n=41) >30ml (n=23) | 3/80 | 0/85 |
| Acosta-Olivo (2024) | 150 ± 108 | 144 ± 90 | NR | NR | NR | NR | NR | NR | NR | NR |
| Atallah (2021) | 96.0 ± 33.6 | 117.0 ± 52.5 | NR | NR | NR | NR | NR | NR | NR | NR |
| do Nascimento (2019) | 150.0 ± 32.5 | 140.0 ± 25.8 | 13.0 ± 13.0 | 14.0 ± 11.0 | NR | NR | NR | NR | 4/49 | 4/47 |
| Gandhi (2023) | 240 ± 60 | 240 ± 60 | NR | NR | NR | NR | NR | NR | NR | NR |
| Han (2024) | 189.0 ± 74.7 | 209.0 ± 75.07 | NR | NR | 22/78 | 22/78 | 250.0 ± 172.2 | 251.0 ± 113.1 | 0/78 | 1/78 |
| Hasan (2020) | 82.2 ± 12.1 | 79.1 ± 19.3 | NR | NR | NR | NR | 63.5 ± 12.9 | 67.5 ± 13.6 | 0/192 | 1/198 |
| Kannan (2025) | 224.4 ± 98.0 | 224.4 ± 106.5 | NR | NR | NR | NR | NR | NR | NR | NR |
| Khalid (2023) | 127.0 ± 47.0 | 120.0 ± 34.0 | NR | NR | 17/39 | 18/39 | 121.0 ± 76.0 | 96.0 ± 32.0 | 1/39 | 5/39 |
| Kunakornsawat (2019) | 2g: 151  1g: 136 | 156 | NR | NR | NR | NR | 2g: 450.0  1g: 470.0 | 440 | NR | NR |
| Mirzashahi (2017) | NR | NR | NR | NR | NR | NR | NR | NR | 10/193 | 5/187 |
| Mohammadhoseini (2024) | NR | NR | NR | NR | NR | NR | NR | NR | NR | NR |
| Morgan (2024) | NR | NR | NR | NR | 6/21 | 5/21 | 519.0 ± 107.9 | 310.2 ± 90.2 | NR | NR |
| Mulpur (2024) | 70.2 ± 16.4 | 71.1 ± 21.9 | NR | NR | NR | NR | NR | NR | 2/507 | 5/515 |
| O'Toole (2021) | 192.0 ± 78.0 | 198.0 ± 90.0 | NR | NR | NR | NR | NR | NR | 29/481 | 46/499 |
| Rathore (2025) | NR | NR | NR | NR | NR | NR | NR | NR | NR | NR |
| Saba (2025) | THA: 109.0 ± 42.1  TKA: 108.0 ± 36.4 | THA: 109.0 ± 38.8  TKA: 105.0 ± 33.1 | NR | NR | NR | NR | NR | NR | 3/455 | 2/484 |
| Salimi (2021) | >180 mins (n=63) | >180 mins (n=67) | NR | NR | 19/187 | 23/188 | 486.0 | 460.0 | 7/187 | 6/188 |
| Singh (2019) | NR | NR | 3.0 ± 0.5 | 3.5 ± 0.5 | NR | NR | NR | NR | NR | 2/50 |
| Tubaki (2013) | NR | NR | NR | NR | NR | NR | Inst.: 230.0 ± 85.0  Uninst.: 35.0 ± 12.0 | Inst.: 220.0 ± 78.0  Uninst.: 30.0 ± 10.0 | 6/433 | 6/474 |
| Wang (2025) | NR | NR | 9.5 ± 5.4 | 8.9 ± 4.6 | NR | NR | 681.3 ± 425.5 | 688.6 ± 424.0 | NR | NR |
| Yusuf (2026) | NR | NR | NR | NR | NR | NR | NR | NR | 5/52 | 6/52 |

**Table S8.** Reported adverse events

| **Author (year)** | **Pulmonary embolism, n/N** | | **Urinary tract infection, n/N** | | **Dural tear, n/N** | | **Non-union, n/N** | | **Wound dehiscence, n/N** | | **Abscess, n/N** | | **Other recorded adverse events (n)** | |
| --- | --- | --- | --- | --- | --- | --- | --- | --- | --- | --- | --- | --- | --- | --- |
|  | **Vanc** | **Control** | **Vanc** | **Control** | **Vanc** | **Control** | **Vanc** | **Control** | **Vanc** | **Control** | **Vanc** | **Control** | **Vanc** | **Control** |
| Abuzaiter (2023) | 1/80 | 2/85 | 0/80 | 0/85 | NR | NR | NR | NR | NR | NR | NR | NR | None | Myocardial infarction (1) |
| Acosta-Olivo (2024) | NR | NR | NR | NR | NR | NR | NR | NR | NR | NR | NR | NR | NR | NR |
| Atallah (2021) | NR | NR | NR | NR | NR | NR | NR | NR | NR | NR | NR | NR | NR | NR |
| do Nascimento (2019) | NR | NR | 11/49 | 10/47 | 3/49 | 3/47 | NR | NR | NR | NR | NR | NR | Bronchopneumonia (2) | Bronchopneumonia (6) |
| Gandhi (2023) | 0/44 | 1/44 | NR | NR | NR | NR | NR | NR | NR | NR | NR | NR | Impaired fracture healing (10)  Bone lysis around implant (1) | Bone lysis around implant (7)  Impaired fracture healing (7)  Pain without weight bearing (3)  Local erythema (2)  Local swelling (2)  Local rise of temperature (2)  Pus drainage (2)  Implant loosening (2)  Fistula/sinus/wound breakdown (1)  Fever (1) |
| Han (2024) | NR | NR | NR | NR | NR | NR | NR | NR | NR | NR | NR | NR | NR | NR |
| Hasan (2020) | NR | NR | NR | NR | NR | NR | NR | NR | NR | NR | 0/192 | 1/198 | NR | NR |
| Kannan (2025) | 1/552 | 0/551 | 1/552 | 3/551 | NR | NR | NR | NR | NR | NR | 1/552 | 0/551 | Serious adverse events (49):  General (29)  Neurological (10)  Infection (7)  Vascular (5)  Death (5)  Hearing loss (4)  Gastrointestinal/psychiatric (2) | Serious adverse events (65):  General (46)  Neurological (14)  Infection (7)  Hearing loss (7)  Death (6)  Cardiorespiratory/vascular (6) Renal/immune (2) |
| Khalid (2023) | NR | NR | NR | NR | NR | NR | NR | NR | NR | NR | NR | NR | NR | NR |
| Kunakornsawat (2019) | NR | NR | NR | NR | NR | NR | NR | NR | NR | NR | NR | NR | Pseudoarthrosis (1) | NR |
| Mirzashahi (2017) | NR | NR | NR | NR | NR | NR | NR | NR | NR | NR | NR | NR | NR | NR |
| Mohammadhoseini (2024) | NR | NR | NR | NR | NR | NR | NR | NR | NR | NR | NR | NR | NR | NR |
| Morgan (2024) | NR | NR | NR | NR | NR | NR | NR | NR | 0/21 | 2/21 | NR | NR | 0 | Cerebrospinal fluid leak (2)  Wound discharge (4) |
| Mulpur (2024) | NR | NR | NR | NR | NR | NR | NR | NR | NR | NR | 20/507 | 13/515 | Persistent wound drainage (47)  Delayed stitch removal (36) Periprosthetic fracture (2) | Delayed stitch removal (36)  Persistent wound drainage (26)  Sepsis (1)  Deep vein thrombosis (1) |
| O'Toole (2021) | NR | NR | NR | NR | NR | NR | 23/481 | 18/499 | 17/481 | 23/499 | NR | NR | Serious adverse events (42) | Serious adverse events (46) |
| Rathore (2025) | NR | NR | NR | NR | NR | NR | 12/64 | 8/64 | NR | NR | NR | NR | NR | NR |
| Saba (2025) | NR | NR | NR | NR | NR | NR | NR | NR | 11/455 | 11/484 | 0/455 | 2/484 | NR | NR |
| Salimi (2021) | NR | NR | 3/187 | 2/188 | 11/187 | 12/188 | NR | NR | NR | NR | NR | NR | NR | NR |
| Singh (2019) | NR | NR | NR | NR | NR | NR | 1/50 | 5/50 | NR | NR | NR | NR | Graft rejection (1)  Fever (1)  Abnormal swelling/discharge (2) | Graft rejection (1)  Fever (6)  Abnormal swelling/discharge (5)  Chronic fistula (2) |
| Tubaki (2013) | NR | NR | NR | NR | NR | NR | NR | NR | NR | NR | NR | NR | NR | NR |
| Wang (2025) | NR | NR | NR | NR | 15/357 | 12/348 | NR | NR | NR | NR | NR | NR | Screw loosening (59) | Screw loosening (53)  Pneumonia (1) |
| Yusuf (2026) | NR | NR | NR | NR | NR | NR | NR | NR | NR | NR | NR | NR | NR | NR |

**Table S9.** Sensitivity analyses for sparse-event primary outcomes

| **Method** | **Overall SSI, RR or OR (95% CI)** | ***P-*value** | **Superficial SSI, RR or OR (95% CI)** | ***P-*value** | **Deep SSI, RR or OR (95% CI)** | ***P-*value** | **Gram-positive SSI, RR or OR (95% CI)** | ***P-*value** |
| --- | --- | --- | --- | --- | --- | --- | --- | --- |
| Mantel-Haenszel RR with continuity correction of 0.5 (primary) | 0.79 (0.64–0.99) | .04 | 0.91 (0.63–1.33) | .62 | 0.78 (0.57–1.07) | .12 | 0.53 (0.35–0.79) | .002 |
| Peto OR without continuity correction | 0.68 (0.51–0.92) | .01 | 0.66 (0.37–1.19) | .17 | 0.72 (0.48–1.08) | .12 | 0.50 (0.34–0.72) | <.001 |
| Mantel-Haenszel RR with treatment–arm continuity correction | 0.79 (0.64–0.99) | .04 | 0.91 (0.63–1.33) | .62 | 0.78 (0.57–1.07) | .12 | 0.54 (0.37–0.80) | .002 |
| Generalized linear mixed model OR (exact binomial) | 0.68 (0.49–0.96) | .03 | 0.66 (0.35–1.25) | .20 | 0.72 (0.49–1.06) | .10 | 0.48 (0.32–0.72) | <.001 |
| Mantel–Haenszel RR excluding zero-event studies | 0.80 (0.65–1.00) | .047 | 0.98 (0.67–1.44) | .92 | 0.81 (0.58–1.11) | .19 | 0.61 (0.40–0.92) | .02 |

**Table S10.** Low risk of bias sensitivity analyses for primary outcomes

| **Method** | **Overall SSI, RR or OR (95% CI)** | ***P-*value** | **Superficial SSI, RR or OR (95% CI)** | ***P-v*alue** | **Deep SSI, RR or OR (95% CI)** | ***P-*value** | **Gram-positive SSI, RR or OR (95% CI)** | ***P-v*alue** |
| --- | --- | --- | --- | --- | --- | --- | --- | --- |
| Mantel-Haenszel RR with continuity correction of 0.5 (primary) | 0.80 (0.62–1.02) | .071 | 1.06 (0.70–1.62) | .775 | 0.67 (0.48–0.94) | .019 | 0.57 (0.37–0.90) | .016 |
| Peto OR without continuity correction | 0.75 (0.58–0.96) | .025 | 0.99 (0.59–1.64) | .966 | 0.62 (0.41–0.94) | .024 | 0.53 (0.34–0.83) | .005 |
| Mantel-Haenszel RR with treatment-arm continuity correction | 0.80 (0.62–1.02) | .071 | 1.06 (0.70–1.62) | .775 | 0.67 (0.48–0.94) | .019 | 0.57 (0.37–0.90) | .017 |
| Generalized linear mixed model OR (exact binomial) | 0.74 (0.58–0.96) | .025 | 1.01 (0.67–1.53) | .964 | 0.62 (0.44–0.86) | .005 | 0.52 (0.33–0.83) | .005 |
| Mantel-Haenszel RR excluding zero-event studies | 0.78 (0.61–1.00) | .054 | 1.06 (0.70–1.62) | .775 | 0.67 (0.48–0.95) | .023 | 0.62 (0.38–1.00) | .051 |

**Table S11.** Vancomycin powder-only sensitivity analyses for primary outcomes

| **Outcome** | **RR (95% CI)** | ***P*-value** | **I²** | **τ^2^** | **Q** |
| --- | --- | --- | --- | --- | --- |
| **Overall SSI** | 0.76 (0.59–0.99) | .039 | 22.8 | 0 | 19.44 |
| **Superficial SSI** | 0.55 (0.26–1.15) | .113 | 17.6 | 0.305 | 8.49 |
| **Deep SSI** | 0.84 (0.56–1.25) | .384 | 12.4 | 0.048 | 11.41 |
| **Gram-positive SSI** | 0.49 (0.32–0.74) | <0.001 | 0 | 0 | 4.95 |

**Table S12.** Meta-regression results. β, regression coefficient; SE, standard error; QE, test of moderators; df, degrees of freedom; I², residual heterogeneity

| **Outcome** | **Moderator** | **β (SE)** | ***P*-value** | **Residual I², %** | **QE (df)** | ***P* for QE** |
| --- | --- | --- | --- | --- | --- | --- |
| Overall SSI | Vancomycin dose | 0.3885 (0.2583) | .1326 | 0.00 | 19.19 (18) | .3802 |
|  | Instrumentation status | 0.0489 (0.2589) | .8502 | 0.00 | 24.55 (21) | .2671 |
|  | Mean age | 0.0277 (0.0129) | .0317 | 0.00 | 19.97 (21) | .5230 |
|  | Male proportion | -2.041 (0.8095) | .0117 | 0.00 | 18.23 (21) | .6345 |
|  | Diabetic proportion | -0.5454 (1.4489) | .7066 | 0.00 | 24.36 (20) | .2271 |
|  | Smoker proportion | -0.3811 (0.7442) | .6086 | 0.00 | 23.26 (17) | .1411 |
|  | Body mass index | 0.0732 (0.0677) | .2796 | 4.23 | 11.04 (9) | .2727 |
|  | Operative time | 0.0046 (0.0034) | .1769 | 0.00 | 10.09 (11) | .5222 |
|  | Control-group SSI risk | -0.0559 (0.0233) | .0164 | 1.08 | 18.91 (21) | .5908 |

**Table S13.** Risk of bias assessment. Cochrane Risk of Bias 2 (RoB 2) for randomized studies, reported by individual domain and overall risk of bias.

**Table S14.** Summary of findings and certainty of evidence assessment. GRADE assessment for each primary outcome in the present review.

**Figure S1.** Forest plot for Gram-positive cocci

**Figure S2.** Forest plot for MRSA

**Figure S3.** Forest plot for Gram-negative SSI

**Figure S4.** Forest plot for reoperation

**Figure S5.** Forest plot for abscess

**Figure S6.** Forest plot for wound dehiscence

**Figure S7.** Forest plot for mortality

**Figure S8.** Forest plot for hospital length of stay

**Figure S9.** Leave-one-out analysis for overall SSI

**Figure S10.** Leave-one-out analysis for Gram-positive SSI

**Figure S11.** Leave-one-out analysis for Gram-positive cocci

**Figure S12.** Baujat plot for overall SSI

**Figure S13.** Baujat plot for Gram-positive SSI

**Figure S14.** Baujat plot for deep SSI

**Figure S15.** Baujat plot for superficial SSI

**Figure S16.** Subgroup analysis of overall SSI by vancomycin dose

**Figure S17.** Subgroup analysis of Gram-positive SSI by vancomycin dose

**Figure S18.** Subgroup analysis of deep SSI by vancomycin dose

**Figure S19.** Subgroup analysis of superficial SSI by vancomycin dose

**Figure S20.** Subgroup analysis of Gram-negative SSI by vancomycin dose

**Figure S21.** Subgroup analysis of reoperation by vancomycin dose

**Figure S22.** Subgroup analysis of abscess by vancomycin dose

**Figure S23.** Subgroup analysis of wound dehiscence by vancomycin dose

**Figure S24.** Subgroup analysis of mortality by vancomycin dose

**Figure S25.** Subgroup analysis of overall SSI by instrumentation status

**Figure S26.** Subgroup analysis of Gram-positive SSI by instrumentation status

**Figure S27.** Subgroup analysis of deep SSI by instrumentation status

**Figure S28.** Subgroup analysis of superficial SSI by instrumentation status

**Figure S29.** Subgroup analysis of Gram-negative SSI by instrumentation status

**Figure S30.** Subgroup analysis of overall SSI by surgical site

**Figure S31.** Subgroup analysis of Gram-positive SSI by surgical site

**Figure S32.** Subgroup analysis of deep SSI by surgical site

**Figure S33.** Subgroup analysis of superficial SSI by surgical site

**Figure S34.** Subgroup analysis of Gram-negative SSI by surgical site

**Figure S35.** Subgroup analysis of overall SSI by surgical specialty

**Figure S36.** Subgroup analysis of deep SSI by surgical specialty

**Figure S37.** Subgroup analysis of superficial SSI by surgical specialty

**Figure S38.** Subgroup analysis of overall SSI by SSI definition

**Figure S39.** Subgroup analysis of Gram-positive SSI by SSI definition

**Figure S40.** Subgroup analysis of deep SSI by SSI definition

**Figure S41.** Subgroup analysis of superficial SSI by SSI definition

**Figure S42.** Trial sequential analysis for overall SSI

**Figure S43.** Trial sequential analysis for Gram-positive SSI

**Figure S44.** Trial sequential analysis for deep SSI

**Figure S45.** Trial sequential analysis for superficial SSI

**Figure S46: Meta-regression for overall SSI**

**(A)** Meta-regression plot showing the relationship between mean cohort age and overall incidence of surgical site infection (SSI). **(B)** Meta-regression plot showing the relationship between male cohort proportion and overall incidence of SSI. **(C)** Meta-regression plot showing the relationship between control-group SSI risk and overall incidence of SSI. Circles represent individual trial cohorts with sizes proportional to study weight. Solid lines indicate the pooled regression slope; dashed lines indicate 95% CIs. RR, risk ratio.

**C**

**Figure S47.** Funnel plot for overall SSI

**Figure S48.** Funnel plot for Gram-positive SSI

**Figure S49.** Funnel plot for deep SSI

**Figure S50.** Funnel plot for superficial SSI
